# Connecting the dots across time: reconstruction of single-cell signalling trajectories using time-stamped data

**DOI:** 10.1098/rsos.170811

**Published:** 2017-08-23

**Authors:** Sayak Mukherjee, David Stewart, William Stewart, Lewis L. Lanier, Jayajit Das

**Affiliations:** 1 Battelle Center for Mathematical Medicine, Research Institute at the Nationwide Children's Hospital, 700 Children's Drive, OH 43205, USA; 2 Department of Pediatrics, The Ohio State University, Columbus, OH 43210, USA; 3 Department of Physics, The Ohio State University, Columbus, OH 43210, USA; 4 Department of Statistics, The Ohio State University, Columbus, OH 43210, USA; 5 Department of Biophysics Program, The Ohio State University, Columbus, OH 43210, USA; 6 Department of Mathematics, University of Iowa, Iowa City, IA 52242, USA; 7 Department of Microbiology and Immunology, University of California, San Francisco, San Francisco, CA 94143, USA; 8 Institute of Bioinformatics and Applied Biotechnology, Electronic City Phase I, Bangalore, 560100 India

**Keywords:** single-cell signalling kinetics, flow cytometry, mass cytometry, trajectory reconstruction, invariants, pair-matching

## Abstract

Single-cell responses are shaped by the geometry of signalling kinetic trajectories carved in a multidimensional space spanned by signalling protein abundances. It is, however, challenging to assay a large number (more than 3) of signalling species in live-cell imaging, which makes it difficult to probe single-cell signalling kinetic trajectories in large dimensions. Flow and mass cytometry techniques can measure a large number (4 to more than 40) of signalling species but are unable to track single cells. Thus, cytometry experiments provide detailed time-stamped snapshots of single-cell signalling kinetics. Is it possible to use the time-stamped cytometry data to reconstruct single-cell signalling trajectories? Borrowing concepts of conserved and slow variables from non-equilibrium statistical physics we develop an approach to reconstruct signalling trajectories using snapshot data by creating new variables that remain invariant or vary slowly during the signalling kinetics. We apply this approach to reconstruct trajectories using snapshot data obtained from *in silico* simulations, live-cell imaging measurements, and, synthetic flow cytometry datasets. The application of invariants and slow variables to reconstruct trajectories provides a radically different way to track objects using snapshot data. The approach is likely to have implications for solving matching problems in a wide range of disciplines.

## 1. Introduction

Tracking signalling events in single cells is a key step towards understanding single-cell response mechanisms. Signalling events are orchestrated by a large number of intercellular molecular species that transmit information pertaining to changes in the extracellular environment to the cell nucleus [1]. Single-cell responses are often influenced by the geometry of multidimensional temporal trajectories describing time evolution of single-cell protein abundances. For example, in human cancer cell lines, fold change in the abundance of the protein phosphorylated Erk (or pErk) as opposed to the absolute value of pErk abundance regulates single-cell growth responses [2]. In general, the signalling kinetics of many molecular species could affect cell decision processes. However, our understanding regarding the link between the geometry of signalling kinetic trajectories and single-cell responses are often limited to few [1–3] molecular species. This is because spectral overlap between fluorescent dyes and photo-toxicity induced by excited fluorophores [3] make it challenging to track a large number (more than 3) of molecular species in live-cell imaging experiments. Flow cytometry [4,5] and recently developed mass cytometry experiments [4,5] can assay 4 to more than 40 proteins simultaneously in single cells at multiple times, but it is not possible to track single cells in these experiments. Is it possible to reconstruct single-cell trajectories, even approximately, from such time-stamped snapshot data? An affirmative answer to this question will be valuable for analysing signalling mechanisms or calculation of autocorrelation functions [6] for extracting relevant time scales and inference of signalling networks [7].

**Figure 1.**
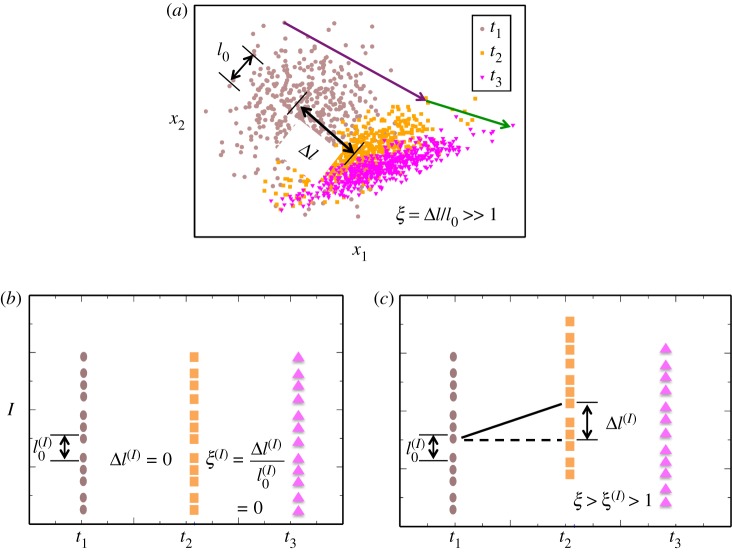
Matching single cells across time using invariants and slow variables. (*a*) Schematic depiction of time-stamped cytometry data showing single cells against copy numbers (denoted by *x*_1_ and *x*_2_) of two signalling species at three different time points *t*_1_ < *t*_2_ < *t*_3_. When the average distance (*l*_0_) between the cells in the *x*_1_–*x*_2_ plane is smaller than the average distance travelled (Δ*l*) in a time interval (e.g., *t*_2_ − *t*_1_) by the cells in the same plane or $x=\Delta l/l_{0}\gg 1$, matching the cells across time is non-trivial due to multiple possibilities. Whereas when *ξ* < 1, connecting the cells could be as straightforward as finding the nearest neighbour. The arrows show a correct trajectory. (*b*) We cast the matching problem in (*a*) in the manifold for a new variable (*I*) constructed from the measured variables (e.g. *x*_1_, *x*_2_). *I* satisfies two conditions: (i) *I* does not change (invariant) or changes substantially slowly (slow variable) in individual cells compared with the original variables or $\Delta l^{\left(I\right)}\ll \Delta l$ and (ii) *I* varies between single cells at any time point. In this situation, $\Delta l^{\left(I\right)}/{l_{0}}^{\left(I\right)}=\xi^{\left(I\right)}\ll 1$ and connecting the cells across time becomes straightforward. When *I* is an invariant, Δ*l*^(*I*)^ *=* 0 and the pairing of cells across time is exact. (*c*) In many cases, *I* will be a slow variable resulting in *ξ*^(*I*)^ > 1. However, even in such cases we find, *ξ*^(*I*)^ < *ξ*, and a cost function is employed to approximately pair single cells across time.

Tracking multiple objects across time using time-stamped data is a common problem encountered in diverse research areas ranging from tracking single molecules in live cells [8] to fluid particles in turbulent flows [9] to birds flying in a flock [10] and to tracking individuals in surveillance videos [11]. The difficulty in tracking individual objects in these problems is characterized by a dimensionless parameter *ξ* = Δ*l*/*l*_0_, where Δ*l* is the average distance an object moves between two successive time recordings and *l*_0_ (=*ρ*^−1/*d*^) is the average object-to-object distance for the objects distributed in *d* dimensions with a density *ρ* [9,12] (figure 1). When ξ≪1 connecting objects across time is straightforward, whereas when ξ≫1 matching objects across time becomes ambiguous. In the later scenario, two types of methods are used to generate solutions—either by optimizing cost functions that are often constructed in an empirical manner [9], or by evaluating probabilities for different matching configurations by estimating parameters in an underlying model [12]. The success of the first method depends on stumbling upon a good cost function and the second method requires intensive computation for the evaluation of the likelihood function and estimation of the model parameters.

Signalling processes usually involve a large number of molecular species (tens to hundreds). Depending on the available antibodies, cytometry techniques assay 4 to more than 40 molecular species in 10^3^–10^6^ cells at chosen time points where the abundances of the marker species have changed appreciably [13]. Thus, the time-stamped data collected in cytometry experiments are almost always in the ξ≫1 range. In addition, involvement of a large number of molecular species in generating a signalling response demands analysis of the kinetic trajectories in high dimensions (*d* > 3). This can create computational road blocks regarding parameter estimation of an underlying model or particle filters using Markov chain Monte Carlo-based algorithms [12,14], which have been extensively used for tracking fluid particles (spatial dimension, *d* = 2) or flocking birds (spatial dimension, *d* = 3). The reconstruction using cytometry data is further complicated as, unlike the above examples, the same objects (i.e. single cells) are not assayed at successive time points.

A fundamentally different way of approaching this problem would be to use the measured variables to construct new variables (*I* in figure 1) that do not change (Δ*l*^(*I*)^ = 0) or change more slowly (Δ*l*^(*I*)^ → 0) with time, while still varying appreciably between the objects at a fixed time point. Thus, when the density of the objects (*ρ*^(*I*)^) in the *d*_(*I*)_ dimensional space of the new variables *I* is small (i.e. large $l_{0}^{\left(I\right)}={\left(1\text{/}\rho^{\left(I\right)}\right)}^{1/{\text{d}}_{I}}$), the matching problem posed in terms of *I* will result in a substantial reduction in the parameter $\xi^{\left(I\right)}(=\Delta l^{\left(I\right)}\text{/}l_{0}^{\left(I\right)})$, which can even fall in the range, $\xi^{\left(I\right)}\ll 1$, where tracking objects is straightforward. In addition, when $\xi \gg \xi^{\left(I\right)}>1$, tracking objects in *I* becomes more amenable to the standard techniques [12,14] due to the lower dimensionality of the manifold and the smaller value of ξ^(*I*)^. However, it is often difficult to construct such conserved or slow variables for the problem in hand. In physical systems, conservation laws (e.g. conservation of energy) [15,16], breakdown of continuous symmetry (e.g. Goldstone modes) [15,16] or the presence of a critical point [15,16] can bring conserved and slow variables into existence. However, direct application of such principles in cell signalling processes is not obvious.

Here, we developed a framework for matching untagged single cells across time by constructing conserved and slow variables using abundances of molecular species that follow biochemical signalling kinetics. The calculations of the new variables do not require any parameter estimation, and thus avoid computational difficulties usually encountered in matching problems. We constructed two invariants for an ideal kinetics where the signalling kinetics is described by a closed system of first-order reactions. One of the invariant variables is based on the conservation of total number of molecular species, and the other one involves scaling the measured abundances by a particular function of the covariance matrix. These invariants turn into slow variables or remain as invariants for more general biochemical reactions that include external fluxes, involve second-order or higher order reactions, and contain stochastic fluctuations [17]. The slow variables and invariants, constructed from the measured species abundances, allowed us to connect a single cell with a ‘sister cell’ at a later time whose species abundances are reasonably close to the correct cell partner. We validate the above formalism by reconstructing trajectories using published live-cell imaging data [18]. Lastly, we apply our framework for reconstructing signalling trajectories between successive time points in a synthetic flow cytometry dataset.

## 2. Results

### 2.1. Development of the framework

In this section, we describe the development of the framework and then evaluate the framework on a range of signalling models based on deterministic linear, deterministic nonlinear and stochastic reactions.

#### 2.1.1. Determination of invariants in an ideal system

We constructed two invariants, *I*_T_ and *I*_M_, for an ideal system of biochemical reactions. The invariants do not change with time but vary between single cells at a particular time. The ideal system satisfies the following conditions: (i) the reaction propensities are linear functions of the copy numbers (or abundances) of the molecular species, (ii) the reaction rates are time independent and there is no external in- (or out-)flux of molecules and (iii) the kinetics does not include any intrinsic noise fluctuations. The mass action kinetics of the biochemical reactions are described as an autonomous system of linear first-order ordinary differential equations (ODEs). Consider an ensemble of *N* number of single cells where a single cell (indexed by *α*) contains *n* different molecular species (indexed by *i*) with abundances $\left\{x_{i}^{\left(\alpha \right)}\right\}$. Any pair of molecular species, *i* and *j*, in an individual cell (say, the *α*th cell) react following the above conditions, and the propensity for the reaction *j* → *i* (or *i* → *j*) is given by $M_{i}^{j}x_{j}^{\left(\alpha \right)}(\text{or}\;M_{j}^{i}x_{i}^{\left(\alpha \right)})$. The reaction rates $M_{i}^{j}$ and $M_{j}^{i}$ are always positive and constant as long as, *i* ≠ *j*. In our notation scheme, the (*i*,*j*) element of the *M* matrix, $M_{i}^{j}$, is associated with a reaction, *j* (superscript index) → *i* (subscript index). Vanishing values for both $M_{i}^{j}$ and $M_{j}^{i}$ would imply the absence of any reaction between the species *i* and *j*. In addition, the elements of the *M* matrix do not depend on the cell index implying that the signalling reactions occur with the same rates in individual cells.

The species abundances in individual cells follow a mass-action linear kinetics described by a set of coupled first-order linear ODEs,

2.1 $$\frac{\text{d}x_{i}^{\left(\alpha \right)}}{\text{d}t}=\sum_{j=1}^{n}M_{i}^{j}x_{j}^{\left(\alpha \right)}.$$

As the elements of the *M* matrix do not depend on time explicitly, the above equation represents an autonomous system [19].

The source of variations in species abundances following the kinetics in equation (2.1) are the cell–cell variations in the pre-stimulus condition ($\left\{x_{i}^{\left(\alpha \right)}(t=0)\right\}$) arising due to cell–cell differences in total content of the molecular species and tonic (basal) signalling [20]. These variations are known as extrinsic noise fluctuations [17,21]. In the ideal case, we assume that cytometry experiments can measure all the signalling species abundances in equation (2.1) at any time. Below we describe the invariants, *I*_T_ and *I*_M_.

##### 2.1.1.1. Total abundance (*I*_T_)

If the rates in equation (2.1) are further constrained to satisfy, $\sum_{i=1}^{n}M_{i}^{j}=0$, then the total copy number of the molecular species 1 to *n* remains unchanged over time in a single cell #*α*, i.e.

2.2 $$I_{T}^{\left(\alpha \right)}=\sum_{i=1}^{n}x_{i}^{\left(\alpha \right)}(t)=\sum_{i=1}^{n}x_{i}^{\left(\alpha \right)}(0).$$

An example of the above case could be first-order reactions describing phosphorylation and dephosphorylation of a single protein species preserving the total number of protein molecules. The number conservation is an elementary conservation principle followed by biochemical reactions of first-order or higher order reactions in the absence of any particle exchange with the environment. We will analyse implications of this conservation principle for non-ideal cases in later sections.

##### 2.1.1.2. Magnitude of the scaled abundance vector (*I*_M_)

The magnitude of the scaled abundance vector ${\tilde{x}}^{\left(\alpha \right)}\equiv \{{\tilde{x}}_{1}^{\left(\alpha \right)},{\tilde{x}}_{2}^{\left(\alpha \right)},\ldots ,{\tilde{x}}_{n}^{\left(\alpha \right)}\}$ in a single cell (#*α*) remains unchanged throughout the kinetics from its value at *t* = 0. ${\tilde{x}}_{i}^{\left(\alpha \right)}$ is defined as

2.3 $${\tilde{x}}_{i}^{\left(\alpha \right)}(t_{1})=\sum_{j=1}^{n}{\left[{\left(J(t_{1})\right)}^{-1/2}\right]}_{ij}{x_{j}}^{\left(\alpha \right)}(t_{1}).$$

The derivation is shown in the Material and methods. The elements of the *J* matrix ({*J_ij_*}) in equation (2.3) denote the covariances of the species abundances at any time *t*

2.4 $$J_{ij}(t)=\frac{1}{N}\sum_{\alpha =1}^{N}(x_{i}^{\left(\alpha \right)}(t)-\mu_{i}(t))(x_{j}^{\left(\alpha \right)}(t)-\mu_{j}(t)),$$

and {*μ_i_*} are the mean species abundances

2.5 $$\mu_{i}(t)=\frac{1}{N}\sum_{\alpha =1}^{N}x_{i}^{\left(\alpha \right)}(t).$$

Both {*μ_i_*} and {*J_ij_*} can be calculated from the cytometry snapshot data.

The magnitude of ${\tilde{x}}^{\left(\alpha \right)}$ defined as

$$m^{\left(\alpha \right)}(t)=|{\tilde{x}}^{\left(\alpha \right)}(t)|=\sqrt{\sum_{i=1}^{n}{\left({\tilde{x}}_{i}^{\left(\alpha \right)}(t)\right)}^{2}}$$

does not change with time, i.e.

2.6 $$I_{M}^{\left(\alpha \right)}=m^{\left(\alpha \right)}(t_{1})=m^{\left(\alpha \right)}(t_{2})=m^{\left(\alpha \right)}(t=0),$$

is an invariant of the kinetics. One can physically interpret the transformation in equation (2.3) in the following way. The first-order chemical reactions rotate (or reflect) as well as stretch (or compress) the abundance vector ***x***^(α)^ (*t*) with time. The transformation in equation (2.3) rescales the vector to offset the stretching (or compressing) component. Subsequently, the time evolution of the scaled vector can be described as a pure rotation (or reflection) (see web supplement). As rotation or reflection is an orthogonal transformation, the magnitude of the scaled variable is preserved.

##### 2.1.1.3. Exact matching using invariants

*I*_T_ and *I*_M_ are functions of copy numbers of molecular species, and therefore vary considerably from cell to cell at a given time point. Thus, these invariants create unique ‘tags’ for single cells, and pairing single cells across time then is reduced to matching the same values of invariants in cell populations at two time points. A possible degeneracy can arise when an invariant takes the same value in multiple single cells. For example, single cells (e.g. #*α* and #*β*) with abundances lying in the plane defined $I_{T}^{\left(\alpha \right)}=\sum_{i}{x_{i}}^{\left(\alpha \right)}(0)=\sum_{i}{x_{i}}^{\left(\beta \right)}(0)=I_{T}^{\left(\beta \right)}$ cannot be resolved by *I*_T._ In such cases the invariant $I_{T}^{\left(\alpha \right)}$ will be unable to match these cells across time. This same difficulty holds for the other invariant $I_{M}^{\left(\alpha \right)}$; however, it is less likely to encounter such degeneracy in this case.

#### 2.1.2. Construction of slow variables for non-ideal situations

**Figure 2.**
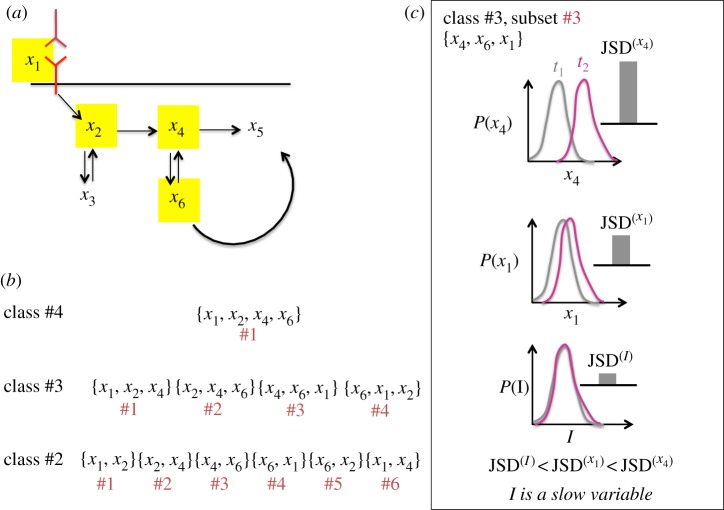
Determination of slow variables or invariants: (*a*) signalling networks are composed of a large number of species; however, only few of them can be assayed. In the schematic network of six different species, only four species (shown with yellow boxes) *x*_1_, *x*_2_, *x*_4_ and *x*_6_ can be measured in experiments. Cytometry experiments measure single-cell abundances of these species (also denoted as {*x_i_*} here). (*b*) 2^4^–4–1 = 11 different subsets of measured species abundances can be constructed. Each of these subsets represents projection of the original data into a manifold spanned by the member species abundances in the subset. The subsets are further divided into classes for our analyses. Each class contains subsets with the same cardinality. For example, class #3 contains all the three species subsets. (*c*) For each subset (e.g. class #3, subset #3), we evaluate the change in the distribution of the species abundances in the cell population using JSD (equation (2.7)). A large change in the distribution of species abundances indicating faster kinetics will result in a larger value of JSD. Whereas if *I* behaves as a slow variable or an invariant, the distribution of *I* in the cell population will go through a small change or no change, respectively. The change in *I* is quantified by JSD^(*I*)^. When JSD^(*I*)^ is smaller than the slowest species in the subset, we denote *I* as a slow variable for that subset. In the example shown in (*c*), *P*(*x*_4_) and *P*(*x*_1_) give rise to the maximum and the minimum values of JSDs in the species subset, i.e. *JSD*^(*x*_1_)^ < *JSD*^(*x*_6_)^ < *JSD*^(*x*_4_)^.

Cell signalling networks often deviate from the ideal kinetics considered above. This occurs due to multiple reasons: (i) it is usually infeasible to assay all the molecular species involved in a signalling network. In that case, the measured species abundances evolve as a non-autonomous system, because the unmeasured species abundances implicitly give rise to time-dependent reaction rates or external fluxes in the kinetics. (ii) The propensities of biochemical signalling reactions are often nonlinear functions of the species abundances. (iii) The copy numbers of molecular species contain stochastic fluctuations arising from intrinsic noise in biochemical reactions. In the presence of the above effects, except few special cases, both *I*_T_ and *I*_M_ cease to behave as invariants of the kinetics. Our investigations (details in §2.1.3.) using simulations of a variety of *in silico* signalling kinetics showed that for specific subsets of species abundances, the variables *I*_M_ and *I*_T_, evolve at a much slower rate compared with the measured species abundances in a time interval. Borrowing from the lexicon of non-equilibrium statistical physics [16], we designate these variables as ‘slow variables’. Next, we discuss our scheme to identify appropriate combination of measured species abundances where *I*_T_ and *I*_M_ behave as slow variables or invariants (also see figure 2).

We used a metric known as the Jensen–Shannon divergence JSD (0 ≤ JSD ≤ ln2) [22,23] to determine slow variables and invariants in our scheme. JSD characterizes the difference between a pair of distributions [23]. JSD vanishes when distributions are identical and increases monotonically as the overlap between the distributions decreases. JSD is defined as

2.7 $$\text{JS}{\text{D}}^{\left(y\right)}=\frac{1}{2}[d_{\mathrm{KL}}(P_{1}(y)∥M)+d_{\mathrm{KL}}(P_{2}(y)∥M)],$$

where *M*(*y*) = 1/2(*P*_1_(*y*) + *P*_2_(*y*)). The *y* superscript in JSD^(*y*)^ denotes the variable in the distributions. Here *d*_KL_ is the Kullback–Leibler divergence [22] between two distributions. When *y* is a discrete variable, *d*_KL_ is given by

$$d_{\mathrm{KL}}(P_{1}(y)∥M)=\sum_{y}P_{1}(y)\ln\left[\frac{P_{1}(y)}{M(y)}\right].$$

When *I *∈ (*I*_T_, *I*_M_) is an invariant of the kinetics, the distribution of *I* in a cell population does not change with time, i.e.

2.8 $$P(I,t_{1})=P(I,t_{2}).$$

An exception to this can arise where *I* varies between individual cells yet the distribution of *I* remains unchanged across time. This can occur when the stochastic kinetics of the chemical reactions is in the steady state where species abundances (and *I*) change across time in individual cells but the distributions of these variables remain time independent. In this situation, the presence of the equality as in equation (2.8) will not imply an existence of a slow variable, *I*. However, such situations can be easily detected from the data by checking if distributions of original variables do not vary across time. Therefore, the JSD between *P*(*I*, *t*_1_) and *P*(*I*, *t*_2_) vanishes when *I* is an invariant, i.e. JSD^(*I*)^ = 0. When *I* does not behave as an invariant, then, JSD^(*I*)^ > 0 (figure 2). However, *I *∈ {*I*_T_, *I*_M_} can still evolve at a slower rate than any of the measured species in the time interval *t*_1_ to *t*_2_. We evaluated this possibility by comparing changes in the JSD values corresponding to the distributions of species abundances and *I* in a cell population in the time interval *t*_1_ to *t*_2_. When *I*_T_ or *I*_M_, evolves slower than any of the individual species (say *x*) abundances in set of species, specifically, when

2.9 $$\text{JS}{\text{D}}^{\left(I_{\text{T}}\;\text{or}\;I_{M}\right)}<\text{JS}{\text{D}}^{\left(x\right)},$$

we denote *I* (*I*_T_ or *I*_M_) as the slow variable for that set of species abundances. In general, one or more of the species abundances in a set of all the measured species will not satisfy the condition in equation (2.9), therefore, *I*_T_ or *I*_M_ cannot be considered as a slow variable for that set. However, for a subset of the measured abundances, *I*_T_ or *I*_M_, could still behave as a slow variable or an invariant. To explore this possibility, we compared the JSD values corresponding to species abundances and *I *∈* *(*I*_M_, *I*_T_) in a time interval *t*_1_−*t*_2_ for all possible subsets that can be constructed using the measured abundances (figure 2). We used a classification scheme for the subsets (figure 2*b*) to describe our results. When *n* number of species abundances are measured, we considered all possible combinations (2^*n*^−*n*−1) of the abundances excluding the singletons.

These subsets were organized into classes where each class (indexed by *k*) contained subsets with the same cardinality (*k*) (figure 2*b*). The cardinality is defined as the number of species abundances in a subset, e.g. for *n* = 14 the class with cardinality *k* = 4 (or class #4) contains ^14^*C*_4_ = 1001 different subsets with each subset composed of four different molecular species abundances. In addition, the subsets within class #*k* were indexed by the integers ({*m*}) 1 to *^*n*^C*_k_. Thus, a particular subset is denoted by a class number *k* and a subset index *m* (figure 2*b*).

##### 2.1.2.1. Matching using slow variables and invariants

We created a cost function *E*, that measures the total Euclidean distance between slow variables in pairs of single cells ({*α*} at *t*_1_, {*β*} at *t*_2_) across time. *E* is defined as

2.10 $$E(\{\beta \})=\sum_{\alpha =1}^{N}{\left(I^{\left(\alpha \right)}(t_{1})-I^{\left(\beta \right)}(t_{2})\right)}^{2}.$$

The ‘sister cells’ constitute the set {*β*^M^} that minimizes *E*. The minimization amounts to a bipartite matching between the set {*α*} and {*β*}; we used an algorithm (see Material and methods) based on sorting with an *O*(*n*ln(*n*)) computation time. A sister cell can be thought of as a partnering cell whose species abundances are not substantially different than that in the correct cell partner.

##### 2.1.2.2. Quantification of quality of matching

We calculated the error in the reconstructed trajectory when a cell *α* at time *t*_1_ was paired with a cell *β* (or the ‘sister cell’) at a later time point *t*_2_ instead of the correct partner cell *β*^′^ at *t*_2_. *β*^′^ is uniquely determined by α for deterministic signalling kinetics in the non-steady state. We defined an error *χ_αβ_* for pairing cell #*α* at time *t*_1_ with cell #*β* at time *t*_2_:

2.11 $$\chi_{\alpha \beta }^{\;}=\sqrt{{\sum_{i=1}^{N}\left(x_{i}^{\left(\beta \right)}-x_{i}^{\left(\beta^{\prime }\right)}\right)}^{2}},$$

where *χ_αβ_* = 0, when *β* = *β*^′^. Note, this metric identifies the cells by the measured species abundances in individual cells, e.g. if the sister cell possesses the same values of the measured abundances as the correct cell partner, the sister cell is identified as the correct match. It is possible that the sister cell contains different values for unmeasured species abundances compared to the correct cell; however, equation (2.11) remains blind to such differences. A small *χ_αβ_* would imply a small difference between the correct partner (*β*′) and the ‘sister cell’ (*β*) for the measured species in the subset. We calculated a distribution of *χ_αβ_* (*P*(*χ*)) for the cell pairs matched using our scheme and compared that with the case when cells were paired randomly across time.

We also calculated the autocorrelation function, *A_ij_*(*t*_1_, *t*_2_), between species *i* at time *t*_1_ and species *j* at time *t*_2_ for the pairings with the correct partner cells and the sister cells. *A_ij_*(*t*_1_, *t*_2_) between the correct cell pairs ({#*α* paired with #*β*′}) is defined as

2.12a $$A_{ij}^{\mathrm{correct}}(t_{1},t_{2})=\frac{1}{N}\sum_{\alpha =1}^{N}(x_{i}^{\left(\alpha \right)}(t_{1})-\mu_{i}(t_{1}))(x_{j}^{\left(\beta^{\prime }\right)}(t_{2})-\mu_{j}(t_{2})).$$

This is compared with the autocorrelation function corresponding to the pairings with the sister cells

2.12b $$A_{ij}^{\mathrm{sister}}(t_{1},t_{2})=\frac{1}{N}\sum_{\alpha =1}^{N}(x_{i}^{\left(\alpha \right)}(t_{1})-\;\mu_{i}(t_{1}))(x_{j}^{\left(\beta (\alpha )\right)}(t_{2})-\mu_{j}(t_{2})),$$

where the sister cell for *α* is indexed by *β*(*α*). The difference between the autocorrelations between the pairings with the correct (equation (2.12*a*)) and the sister cells (equation (2.12*b*)) is calculated by distance Δ*A*(*A*^correct^, *A*^sister^) between the matrices:

2.13 $$\Delta A(A^{\mathrm{correct}},A^{\mathrm{sister}})=\sqrt{\sum_{i=1}^{n}\sum_{j=1}^{n}|A_{ij}^{\mathrm{correct}}-{A_{ij}^{\mathrm{sister}}|}^{2}}.$$

#### 2.1.3. Evaluation of slow modes and quality of matching for model signalling kinetics

Here we investigated the occurrence of slow modes (*I*_M_ and *I*_T_) in subsets of molecular species involved in model biochemical signalling networks as a proof-of-concept for the framework we developed in §2.1.1 and 2.1.2. We studied deterministic and stochastic kinetics in a system of first-order reactions, and the Ras activation signalling network composed of nonlinear biochemical reactions [24]. We assumed that all the signalling species were measured species for the *in silico* networks.

##### 2.1.3.1. Deterministic first-order kinetics

We studied the matching problem for a signalling kinetics described by first-order reaction kinetics composed of 14 different species (electronic supplementary material, figure S1). The ODEs describing the mass action kinetics of all the 14 species is autonomous; however, when subsets of the 14 species are considered, the kinetics is no longer autonomous because the corresponding ODEs contain time-dependent external fluxes arising from the implicit kinetics of the unmeasured species.

**Figure 3.**
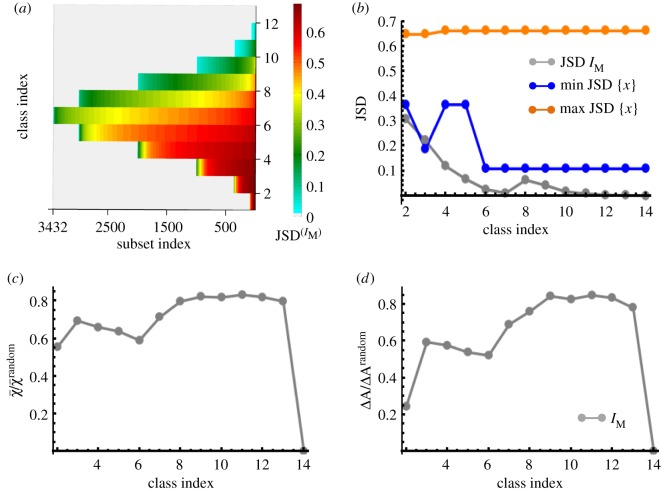
Pairing single cells for the ideal kinetics. (*a*) Heat map for $\text{JS}{\text{D}}^{\left(I_{M}\right)}$. JSD was calculated for 3000 single cells (see Material and methods for details) at time *t*_1_ = 0 and *t*_2_ = 7 min. Other parameters related to the signalling kinetics are shown in the web supplement. (*b*) The minimum values of $\text{JS}{\text{D}}^{\left(I_{M}\right)}$ for each class (grey points). JSD values associated with the fastest (shown in orange) and the slowest species (shown in blue) in the subsets corresponding to the minimum $\text{JS}{\text{D}}^{\left(I_{M}\right)}$ are compared with minimum $\text{JS}{\text{D}}^{\left(I_{M}\right)}$. (*c*) The quality of matching when *I*_M_ was used for matching the cells. The ratio in the average error $\bar{\chi }$ in the matching using *I*_M_ with that for random matching is shown for each of the subsets. $\bar{\chi }$ and ${\bar{\chi }}^{\mathrm{random}}$ are the averages of ${\bar{\chi }}_{\alpha \beta }$ over the single cells pairs matched using our method and random pairing, respectively. $\bar{\chi }<1$ indicates smaller error compared with the random pairing. (*d*) Error in the autocorrelation function (Δ*A*) for the subsets corresponding to the minimum values of $\text{JS}{\text{D}}^{\left(I_{M}\right)}$. Δ*A*^random^ denotes the error in the autocorrelation function for random matching.

*Identification of slow variables and invariants.* We analysed 2^14^ − 14 − 1 = 16 369 different subsets in a time interval where the kinetics is not in the steady state. We acknowledge that the number of possible subsets can become prohibitively large at very large dimensions. We found that for every class (*k* ≥ 2), the subsets produced a wide range of (0 ≤ JSD^(*I*)^ < ln(2)) JSD^(*I*)^ values (figure 3*a*) corresponding to *I* = *I*_T_ (electronic supplementary material, figure S2) and *I* = *I*_M_ (figure 3*a*). Next we analysed if *I*_T_ or *I*_M_ behaved as slow variables or invariants in the subsets that are associated with smaller values of JSD^(*I*)^. The comparison of the minimum values of $\text{JS}{\text{D}}^{\left(I_{M}\right)}$ with JSD values for the fastest and slowest species abundances (figure 3*b*) in the subsets associated with minimum $\text{JS}{\text{D}}^{\left(I_{M}\right)}$ showed that *I*_M_ evolved as a slow variable in most of those subsets. *I*_M_ turned into an invariant when all the 14 species were included in the set (*k* = 14). Similar behaviour was found for *I*_T_ as well (electronic supplementary material, figure S3). The composition of the subsets associated with the minimum values of $\text{JS}{\text{D}}^{\left(I_{M}\;\text{or}\;I_{T}\right)}$ depends on the variable type (*I*_T_ or *I*_M_) and the time interval, as well as on the rate constants of the reaction network.

*Matching using the slow variables.* The cost function in equation (2.10) was minimized using the subsets associated with minimum JSD^(*I*)^ to find the sister cells. The quality of matching was evaluated by calculating the error in matching *χ* (equation (2.11)) for each single-cell–sister-cell pair. We show the results for the pairing carried out using *I*_M_ here. *I*_M_ is an invariant for the subset corresponding to *k* = 14, and minimizing *E* produced an exact matching in that case (figure 3*c*). *I*_M_ turned out to be a slow variable for the other subsets and the quality of matching using *I*_M_ was reasonable (figure 3*c*). The mean *χ*-values ($\bar{\chi }$) for these pairings were 0.6–0.8 times lower than that for random pairing (figure 3*c*). Next, we calculated the autocorrelation function for the matched pairs. Similar to $\bar{\chi }$, errors in the autocorrelation function ranged from very small to moderate values (0.2–0.8 times less) compared with that when the single cells were paired randomly (figure 3*d*). The subsets with smaller number of species show lower error values (figure 3*d*), this could arise due to the sensitivity of the autocorrelation function to small errors in matching in higher dimensions. The quality of matching using *I*_T_ was qualitatively similar to that of *I*_M_ (electronic supplementary material, figure S4). Interestingly, pairing using *I*_T_ produced better agreement with the correct trajectories even when *I*_T_ evolved faster than *I*_M_ (electronic supplementary material, figure S5). This behaviour emphasized the role of the cost function in pair-matching (also see the Discussion).

##### 2.1.3.2. Nonlinear deterministic kinetics

We used an experimentally validated signalling network for Ras activation in T lymphocytes (electronic supplementary material, figure S6) [24]. The network describes enzymatic activation of Ras by two enzymes SOS and Rasgrp1, where SOS-mediated Ras activation contains a positive feedback, i.e. an activated form of Ras or RasGTP induces a higher catalytic rate involving SOS. The deterministic kinetics displays bistability for a range of SOS and Rasgrp1 concentrations. We analysed the non-steady-state kinetics in the model for the parameter values that generate monostable or bistable steady states. The *in silico* model contained 14 different molecular species.

**Figure 4.**
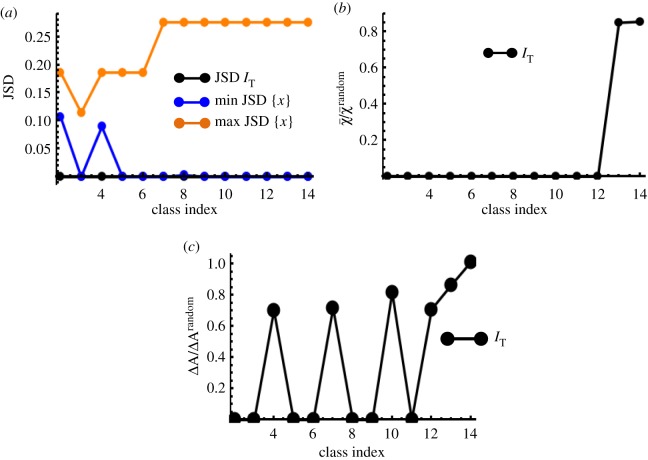
Pairing single cells for non-ideal kinetics. (*a*) The minimum values of $\text{JS}{\text{D}}^{\left(I_{T}\right)}$ for each class (black points) for the deterministic Ras activation kinetics model. We used 3000 single cells across time points *t*_1_ = 100 s and *t*_2_ = 400 s, where the Ras activation displays bistability. $\text{Min}(\text{JS}{\text{D}}^{\left(I_{T}\right)})$ values were compared with the JSD values associated with the fastest (shown in orange) and the slowest species (shown in blue) in the subsets corresponding to $\min(\text{JS}{\text{D}}^{\left(I_{T}\right)})$. *I*_T_ behaves as an invariant or a slow variable for the subsets. (*b*) The quality of matching given by $\bar{\chi }/{\bar{\chi }}^{\mathrm{random}}$ when *I*_T_ was used for matching the cells for the subsets associated with $\min(\text{JS}{\text{D}}^{\left(I_{T}\right)})$ in (*a*). (*c*) Error in the autocorrelation function (Δ*A*) when the cells were matched using *I*_T_ for the stochastic Ras activation kinetics. The subsets used in the matching yielded minimum values of $\text{JS}{\text{D}}^{\left(I_{T}\right)}$ for each class. Note that for exact matching (Δ*A* = 0) *I*_T_ behaved as an invariant.

*Identification of slow variables and invariants.* Similar to the first-order kinetics in the previous section, the JSD^(*I*)^ values corresponding to *I* = *I*_T_ showed large variations across subsets (electronic supplementary material, figure S7). However, a noted difference from the previous example was that *I*_T_ behaved as an invariant for multiple subsets. This is because, even with the nonlinear rates, the total number of certain species is an invariant of the kinetics. For example, the total amount of Rasgrp1 contained in the complexes (free Rasgrp1, Rasgrp1-DAG, Rasgrp1-DAG-RasGDP) remained fixed throughout the kinetics, and the subset {Rasgrp1, Rasgrp1-DAG, Rasgrp1-DAG-RasGDP} produced the minimum $\text{JS}{\text{D}}^{\left(I_{T}\right)}$ (=0) in the class *k* = 3 (figure 4*a*). *I*_M_, in contrast, evolved only as a slow variable in specific subsets (electronic supplementary material, figure S8).

*Matching using the slow variables.* For the subsets where *I*_T_ behaved as an invariant, the matching produced exact alignment. For other subsets corresponding to the minimum values of $\text{JS}{\text{D}}^{\left(I_{T}\right)}$, the pairing was substantially better than that of random matching (figure 4*b*). *I*_M_-guided pairing showed small errors in abundances in the sister cells (electronic supplementary material, figure S9); however, overall pairing using *I*_T_ produced smaller errors.

##### 2.1.3.3. Stochastic signalling kinetics

We simulated stochastic biochemical reactions in the Ras activation signalling kinetics by including intrinsic noise fluctuations. The simulations contained the same variations in the initial abundances used in the investigations for the deterministic kinetics. *I*_T_ evolved as an invariant in subsets that were associated with conservation of the total number of molecular species (electronic supplementary material, figure S10). *I*_T_ also behaved as a slow variable in specific subsets (electronic supplementary material, figure S10*a*). By contrast, *I*_M_ behaved as a slow variable for select subsets (electronic supplementary material, figure S10*b*). Pairing the single cells using *I*_T_ for the subsets associated with minimum values $\text{JS}{\text{D}}^{\left(I_{T}\right)}$ showed reasonably smaller errors in the autocorrelation function compared with that for random pairings for most of the subsets (figure 4*c*). The matching using *I*_M_ showed smaller errors (or *χ*) compared with random pairing in select subsets (electronic supplementary material, figure S11).

### 2.2. Application of the framework for matching in live-cell imaging data

**Figure 5.**
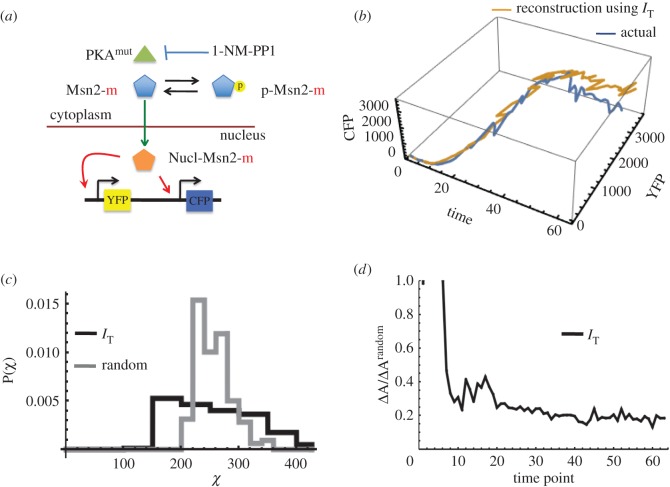
Trajectory reconstruction using live-cell imaging data. (*a*) Schematic diagram for gene activation. (*b*) Reconstruction of a typical kinetics trajectory for CFP and YFP tagged dual reporter for the gene DCS2 in yeast diploid cells. The reconstructed trajectory using *I*_T_ (shown in orange) is compared with the true trajectory (shown in blue). (*c*) Distribution of the quality of alignment *χ* using *I*_T_ is compared to the quality of alignment using random pairing for 159 single-cell trajectories. The reconstructions were carried out between two successive time measurements for 63 time intervals (e.g. 0–2.5 min, 2.5–5 min and so on). (*d*) The ratio of the errors (equation (2.13)) in the autocorrelation function for trajectories reconstructed using *I*_T_ with that for random pairing for the same time intervals in (*c*). Δ*A*/Δ*A*_random_ < 1 for most of the time points indicating better matching using *I*_T_ compared to the random pairing. The data not shown on the graph for the time points between 0 to 10 min produced Δ*A*/Δ*A*^random^ > 1.

We reconstructed single-cell gene expression kinetic trajectories by applying our framework using live-cell imaging data [18]. Data were collected in single yeast cells where the transcription factor Msn2 translocated to the nucleus upon inhibition of protein kinase A by a small molecule, 1-NM-PP1 and activated target fluorescent reporter genes CFP and YFP residing on homologous chromosomes in the diploid yeast cells (figure 5*a*). For our reconstruction, we chose the kinetics of the dual reporter of the gene DCS2 (one of the seven genes activated in the study) induced by a single 40 min pulse of 1-NM-PP1 at a concentration of 690 nM. The activation kinetics of the reporters depended nonlinearly on the Msn2 abundance and also contained intrinsic and extrinsic noise fluctuations [18]. We carried out our reconstruction method by treating the live-cell imaging data as snapshot data, because it allowed us to compare the reconstructed trajectories with the measured single-cell trajectories. We analysed the simultaneous CFP and YFP gene expression kinetics data in 159 single-cell trajectories [18]. At each time point, we removed the single-cell tag from the CFP and YFP expressions data and treated the 159 data points as snapshot data to perform the reconstruction. We reconstructed two-dimensional (CFP and YFP) single-cell trajectories using either *I*_T_ (figure 5*b*) or *I*_M_ (electronic supplementary material, figure S12), and both showed a similar level of agreement between the measured and the reconstructed trajectories. We further quantified the quality of alignment using *P*(*χ*) (figure 5*c*) and autocorrelation functions (figure 5*d* and electronic supplementary material, S12) for each reconstruction. Both indicators revealed lower errors in the reconstruction using *I* compared with random pairing.

### 2.3. Application of the framework for matching single cells using synthetic flow cytometry data

We applied our framework on synthetic flow cytometry data for matching single cells assayed at two successive time points. We used *in silico* or synthetic data instead of measured data from flow cytometry experiments for two main reasons: (i) The synthetic data allowed us to assess the quality of the reconstruction because the correct trajectories are known in the simulations. (ii) We were able to use species abundances in the synthetic data to calculate the slow variables. Single-cell protein abundances are not directly accessible from the fluorescent intensities measured in flow cytometry. However, for many proteins it is possible to quantify single-cell species abundances by calibrating the intensities against a standard curve [25]. The synthetic flow cytometry data were generated at multiple time points by stochastic simulation (details in Material and methods) of the Ras activation network. The species abundances at the unstimulated state (or *t* = 0) in individual cells were chosen from a multivariate normal distribution (Material and methods) to represent cell–cell differences in total protein abundances and tonic signalling. Six molecular species (RasGTP, RasGDP, SOS, RasGRP1, DAG and RasGAP) out of the 14 signalling species involved in the Ras signalling network (electronic supplementary material, figure S6) were recorded in the synthetic data. We used different sets of cell populations to record single-cell species abundances at any two time points in the synthetic data as the same cell populations are not assayed more than once in flow and mass cytometry experiments. We used *I*_T_ instead of *I*_M_ for all our reconstructions here because our previous investigations showed *I*_T_ generated less error in reconstruction compared with *I*_M_ for the Ras activation network. In addition to carrying out reconstruction of trajectories between a pair of successive time points, we addressed the following relevant issues. (i) Is there a specific subset of measured abundances that produces a more accurate reconstruction compared with the other subsets? (ii) How does the error in the reconstruction depend on the size of the time interval between two successive measurements? (iii) How does the reconstruction using *I*_T_ compare against other available methods for matching?

**Figure 6.**
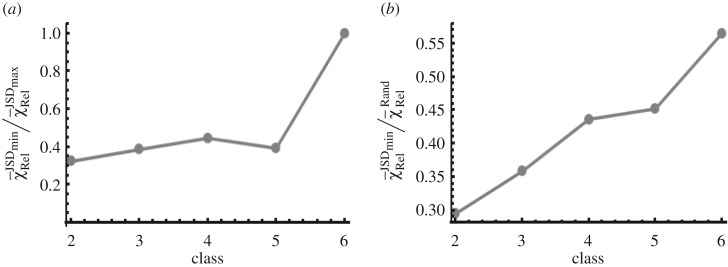
Reconstruction using synthetic flow cytometry data. (*a*) The variation of the ratio of the average relative error ${\bar{\chi }}_{\mathrm{Rel}}^{\mathrm{JS}D_{\min}}/{\bar{\chi }}_{\mathrm{Rel}}^{\mathrm{JS}D_{\max}}$ with the class *k*. The data were measured at *t* = 0 and *t* = 100 s. ${\bar{\chi }}_{\mathrm{Rel}}^{\mathrm{JS}D_{\min}}$ and ${\bar{\chi }}_{\mathrm{Rel}}^{\mathrm{JS}D_{\max}}$ for a class *k* were calculated for the species subsets that generated the smallest and the largest values of $\text{JS}{\text{D}}^{\left(I_{T}\right)}$, respectively. The smaller than 1 values for the ratio indicate that the errors in the subset corresponding to JSD_min_ were smaller on average than that for the subset with the largest JSD in the same class. The subsets the corresponded to JSD_min_ are the following: {RasGDP, RasGAP} for *k* = 2, {RasGDP, RasGTP, RasGAP} for *k* = 3, {RasGDP, RasGTP, RasGAP, DAG} for *k* = 4, {RasGDP, RasGTP, RasGAP, DAG, RasGRP1} for *k* = 5 and {RasGDP, RasGTP, RasGAP, DAG, RasGRP1, SOS} for *k* = 6. The relative error $\chi_{\mathrm{Rel}}^{\alpha \beta }$ between a cell #*α* at *t*_1_ and its matching partner cell# *β* (correct partner cell# *β*′) at *t*_2_ is defined as χRelαβ=∑i=1k(xi(β)−xi(β′))2/|xi(β′)|. We used this definition instead of equation (2.11) to compare between the subsets containing abundances of very different values. The average value of ${\bar{\chi }}_{\mathrm{Rel}}^{\;}$ was calculated by taking the average of $\chi_{\mathrm{Rel}}^{\alpha \beta }$ values for all the single-cell pairs. (*b*) The ratio ${\bar{\chi }}_{\mathrm{Rel}}^{\mathrm{JS}D_{\min}}/{\bar{\chi }}_{\mathrm{Rel}}^{\mathrm{Rand}}$ for the pairing for subsets associated with minimum JSD in (*a*). ${\bar{\chi }}_{\mathrm{Rel}}^{\mathrm{Rand}}$ is the average relative error when the matching was generated using random pairing of the cells. The ratio is less than 1 for all the cases indicating that the reconstruction is better than random reconstructions.

In our investigation, we divided the six measured species into five classes (figure 2 and §2.1.2 for more details) composed of 2, 3, 4, 5 and 6 species. In each class, we determined the subsets that produced the largest and the smallest $\text{JS}{\text{D}}^{\left(I_{T}\right)}$ values. The subset corresponding to the smallest $\text{JS}{\text{D}}^{\left(I_{T}\right)}$gives rise to the slowest *I*_T_ in a class. The subsets corresponding to the lowest $\text{JS}{\text{D}}^{\left(I_{T}\right)}$ values in a time interval also produced the lowest errors in the reconstruction in each class (figure 6*a*). All the reconstructions corresponding to the lowest $\text{JS}{\text{D}}^{\left(I_{T}\right)}$ values faired better than the random pairings (figure 6*b*). These results demonstrate that it is possible to identify a group of species in the cytometry dataset that will generate better reconstruction in a time interval. Moreover, when the interest is in reconstructing trajectories for a particular subset of molecular species, the $\text{JS}{\text{D}}^{\left(I_{T}\right)}$ for the subset can be used to assess the quality of the reconstruction relative to the other combinations of the measured species. This knowledge can help to refine the measurements, e.g. use smaller time intervals (see below) when the $\text{JS}{\text{D}}^{\left(I_{T}\right)}$ value for the subset of interest is not the minimum in the class. Thus, the $\text{JS}{\text{D}}^{\left(I_{T}\right)}$ value provides a valuable metric to quantitatively assess the quality of a trajectory reconstruction in experimental flow cytometry datasets where the correct trajectory is unknown. Using our *in silico* data, we found that the errors become larger as the time interval is increased (electronic supplementary material, figure S13*a*). This is expected as the slow variables start changing appreciably with the increasing magnitude of the time interval resulting in large values of the parameter $\xi_{I}(\gg 1)$. However, the precise answer to the question, if there exists an optimal time interval for cytometry measurements for good trajectory reconstructions needs further investigation (see Discussion). Lastly, we compared our method against a well-known scheme [9] that minimizes the total Euclidean distance between the measured variables at time *t*_1_ and *t*_2_ (electronic supplementary material, figure S13*b*,*c*). Our investigations produced mixed results. For a two-species sub-module our method faired better (electronic supplementary material, figure S13*b*), whereas for a three-species sub-module the quality of reconstruction between the two methods was comparable (electronic supplementary material, figure S13*c*). In a few cases, we found that the Euclidean method produced slightly better reconstructions.

## 3. Discussion

We have developed a framework for matching single cells across time using time-stamped data from flow and mass cytometry experiments. Our approach, based on posing the problem in terms of new variables that remain unchanged or vary slowly with time, is radically different from the existing methods [9,12] employed for solving matching problems. Specifically, unlike other pair-matching algorithms [12], our approach does not require any assumption regarding the underlying reaction kinetics and estimation of model parameters. The use of slow variables and invariants reduces the value of parameter *ξ* making the matching straightforward (when *ξ* < 1) or more amenable to existing computational methods. The application of the framework for *in silico* signalling networks, live-cell imaging data and synthetic flow cytometry data showed excellent to reasonable agreement of the reconstructed trajectories with the correct kinetics. We constructed two new variables from the measured species abundances that served as slow variables or invariants in a wide variety of signalling kinetics involving cell-to-cell variations of species abundances, nonlinear reaction propensities and intrinsic noise fluctuations. One of the new variables (*I*_T_) is the total abundance of a molecular species in a single cell. Early time signalling events usually involve chemical modifications of signalling proteins (e.g. phosphorylation) that do not lead to any change in the total content of the proteins in a single cell. However, the total abundance of a protein at any time can vary from cell to cell over a wide range usually described by a lognormal distribution [26,27]. Thus, the total protein content provides a unique tag that can be used to pair a single cell at any time with a ‘sister’ cell at a later time. In flow and mass cytometry measurements, it is possible to assay the total content of certain signalling proteins (e.g. Erk), which can be used to reconstruct single-cell signalling trajectories.

The other new variable (*I*_M_) was constructed by scaling the measured single-cell abundances by the inverse of the square root of the covariance matrix for species abundances. The reconstruction using *I*_M_ worked better when the signalling kinetics in a time interval was effectively described by a closed system of first-order reactions. As *I*_M_ behaves as a slow variable for a kinetics described by first-order reactions with small stochastic fluctuations and weak external fluxes, it can be used for addressing matching problems in other contexts such as tagged particles in fluid flows [9,12]. We recently developed a method to estimate reaction rates in a system of first-order reactions designed to describe mass cytometry snapshot data in a time interval [7]. The method also determines how well the system of first-order reactions can describe the snapshot data. In the case of a good agreement, we expect *I*_M_ to behave as a slow variable and generate good to reasonable reconstructions. However, the precise relationship between *I*_M_ being a slow variable and the underlying reaction network will require further investigation.

Our investigations of signalling kinetics in non-ideal cases showed that in several cases (electronic supplementary material, figure S5) the reconstructed trajectories using *I*_T_ produced better agreement to the correct kinetics as opposed to *I*_M_. In these cases, the use of *I*_T_ or *I*_M_ reduced the value of the parameter *ξ*; however, *ξ*^(*I*)^ still remained greater than 1, i.e. *ξ*^(*I*)^ > 1. Thus, the cost function *E* (equation (2.10)) played an important role in matching the cells in these situations. The differences in quality of matching between the two slow variables point to the fact that minimizing *E* was better suited for *I*_T_ compared with *I*_M_ in these examples.

The application of our framework to synthetic flow cytometry data showed that $\text{JS}{\text{D}}^{\left(I_{T}\right)}$values, evaluated for different subsets of measured species abundances, can be used to determine groups of species where the reconstruction will produce less errors. The quality of the reconstruction improved as the size of the time intervals between successive measurements was decreased, raising an important question about the existence of an optimal time interval. Similar questions in the context of selecting time points for measuring gene expressions have been dealt with methods based on machine learning where specific cost functions were optimized using finely spaced time measurements in a smaller gene subset [28]. Such approaches might turn out to be helpful for generating trajectory reconstructions using cytometry data where live-cell imaging measurement of a smaller subset of proteins can be used to select time points in cytometry experiments measuring a large number of proteins simultaneously. Another question related to the above issue is if all the cells need to the coherently stimulated in cytometry experiments at time *t* = 0 for good reconstructions. When the time difference in triggering for different batches of cell populations is long enough to produce large values in the parameter $\xi^{\left(I\right)}$, the reconstructions are likely to contain large errors. We also noticed deterioration (electronic supplementary material figure S13*a*) in the reconstruction quality as the kinetics approached the bistable behaviour where the Ras activation changed by a large amount in a very short time interval. The framework is likely to be error prone when such abrupt changes occur in the kinetics.

In recent years, a host of methods have been developed to construct single-cell development trajectories using snapshot data (e.g. WANDERLUST [29], SCUBA [30]) where, unlike the cases dealt with here, the single cells are not ordered temporally. These methods assign a ‘pseudo time’ to the data and then optimize *ad hoc* cost functions to create single-cell trajectories. It is unclear whether those cost functions will render any benefit to the matching problems considered here. For example, one of the cost functions that minimized the cosine distance between single cells [29] will not be able to correctly reconstruct signalling trajectories in a simple example where the kinetics are described by first-order reactions.

The main difficulty in applying the framework developed here is to identify appropriate invariants or slow variables in a general situation. Singer *et al*. [31] used nonlinear independent component analysis for constructing slow variables by analysing stochastic kinetics of dynamical systems in a short time window. This approach determined slow variables that were functions of linear combinations of the observables. When the underlying signalling reactions are known, this approach can help find slow modes in the system by simulating the *in silico* network in short time durations, and these slow variables can then be used to reconstruct single-cell trajectories for cytometry data using our framework. However, the applicability of the approach when the slow modes are complicated nonlinear functions of the measured variables or when only a subset of involved dynamical variables is measured is unclear. In statistical physics [15,16], conservation laws (e.g. conservation of energy, momentum) or symmetry principles help identify such slow modes. Projection operator methods by Zwanzig & Mori [32] provide a formal way to construct variables with slower time scales for a known microscopic dynamics. However, this method requires knowledge of model parameter values (e.g. reaction rates), and the calculations for constructing slow modes could become intractable for a complex system composed of nonlinear interactions such as cell signalling kinetics. A computation intensive step in our framework is to determine specific combinations of species abundances that are associated with low JSD^(*I*)^ values. Mass-cytometry experiments can measure over 40 different proteins and the number of possible subsets in such large dimensions can be prohibitively large. When the signalling reactions are well characterized, selecting a group of species that are connected by mass balance in chemical modifications (e.g. enzymatic conversions or binding–unbinding reactions) could provide a way to identify a core species set with a slow mode. Adding new groups of species using smart sampling techniques [33] to expand this core set would be an exciting future endeavour.

## 4. Material and methods

### 4.1. Derivation of *I*_M_ for the ideal kinetics

Equation (2.1) can be solved analytically to calculate single-cell abundances at any time *t*:

4.1 $$x_{i}^{\left(\alpha \right)}(t)=\sum_{j=1}^{n}{\left[e^{Mt}\right]}_{ij}x_{j}^{\left(\alpha \right)}(0).$$

When the abundances follow the above equation, the average quantities in equations (2.4) and (2.5) at the two time points (*t*_1_ and *t*_2_, *t*_2_ > *t*_1_) are related by

4.2a $$\mu_{i}(t_{2})=\sum_{j=1}^{n}{\left[{\text{e}}^{M(t_{2}-t_{1})}\right]}_{ij}\;\mu_{j}(t_{1})$$

and

4.2b $$J(t_{2})={\text{e}}^{M(t_{2}-t_{1})}J(t_{1})\;{\text{e}}^{M^{T}(t_{2}-t_{1})}.$$

Note that the elements of the *M* matrix cannot be uniquely determined from the above relations because there are *n*^2^ unknown elements in the *M* matrix and equation (4.2*a,b*) provide *n* + *n*(*n* + 1)/2 < *n*^2^ relations between the unknown variables. Therefore, equation (4.1) cannot be used to evolve the abundances in a single cell at time point (at *t*_1_) to a later time point (*t*_2_), and then identify the correct cell from the measurements at *t*_2_. Now, equation (4.2*b*) can be recast as

4.3 $${\text{e}}^{M(t_{2}-t_{1})}={\left[J(t_{2})\right]}^{1/2}Q{\left[J(t_{1})\right]}^{-1/2},$$

where *Q* is any orthogonal matrix, i.e. *QQ*^T^ = *I*, *I* is the identity matrix. This equation contains the clue that if the abundances are scaled appropriately, the time evolution given by equation (2.1) can be described by a rotation or a reflection. We found that if the species abundances at any time are scaled by the inverse of the square root of the covariance matrix (equation (2.4)), then the scaled abundances are related across time points by orthogonal transformations.

Using equation (4.4), we can write equation (4.1) as

4.4 $$x_{i}^{\left(\alpha \right)}(t_{2})=\sum_{j=1}^{n}{\left[{\left[J(t_{2})\right]}^{1/2}Q{\left[J(t_{1})\right]}^{-1/2}\right]}_{ij}x_{j}^{\left(\alpha \right)}(t_{1}),$$

which implies that the scaled variables in equation (2.3) at *t*_1_ and *t*_2_ are related by

4.5 $${\tilde{x}}_{i}^{\left(\alpha \right)}(t_{2})=\sum_{j}Q_{ij}{\tilde{x}}_{j}^{\left(\alpha \right)}(t_{1}).$$

As rotation and reflection preserves the magnitude of a vector, the magnitude (*I*^(*α*)^_M_) of the vector ${\tilde{x}}^{\left(\alpha \right)}\equiv \{{\tilde{x}}_{1}^{\left(\alpha \right)},{\tilde{x}}_{2}^{\left(\alpha \right)},\ldots ,{\tilde{x}}_{n}^{\left(\alpha \right)}\}$ remains unchanged throughout the kinetics from its value at *t* = 0.

### 4.2. Simulation of the *in silico* networks

The mass action deterministic kinetics and the stochastic kinetics for the reactions for the system of first-order reactions and the Ras activation network were simulated using the software package BIONETGEN [34]. The initial species abundances were drawn from a multivariate normal distribution by specifying the average abundances and the covariances. The initial conditions and the parameter values for the reaction networks are provided in the electronic supplementary material, tables S1 and S2, and figures S1 and S6.

### 4.3. Generation of the synthetic flow cytometry data

The kinetics of Ras activation network (electronic supplementary material, figure S6) was simulated using BIONETGEN [34]. Single-cell abundances of six different species (RasGTP, RasGDP, SOS, RasGRP1, DAG and RasGAP) were measured at different times (*t* = 0, 100, 200, 300 and 500 s).

Different batches of 2000 single cells were used for measurements at two successive time points.

### 4.4. Minimization of the cost function *E*

The minimization of *E* involves finding a bipartite graph where a pair of vertices in two subsets (single cells {*α*} at *t*_1_ and single cells {*β*} at *t*_2_) connected with a cost (*I*^(*α*)^ − *I*^(*β*)^)^2^ minimizes the total cost *E*. The graph matching algorithms computes the optimization in time *O*(|*E*|√*V*) ≈ *O*(*n*^2^) [35]. However, in our case we can use sorting to compute this in *O*(*n*ln(*n*)) time. This is achieved by changing the cost function for a pairing #*α*, #*β* to (*I*^(*α*)^ − *I*^(*β*)^)^2^ + *ϵ*(*t*_2_ −*t*_1_)^2^, where, ϵ → 0. Note, this is the Euclidean distance between the cells in the *I*−*t* plane, thus minimizing the cost function *E* amounts to joining these points (or single cells) in the *I*−*t* plane by non-intersecting lines. As the cells in {*α*} (or {*β*}) have the same *t* coordinate *t* = *t*_1_ (or *t*_2_), the configuration with the non-intersecting lines keeps the same ascending (or descending) order in *I* for the cells in {*α*} and {*β*}. Therefore, first, we sorted the {*α*} and the {*β*} cells in arrays where cells were arranged in ascending order of *I*, and then in the sorted arrays, we connected cells with the same array index. A pseudo-code is provided in the electronic supplementary material. The Mathematica codes are available at http://planetx.nationwidechildrens.org/~jayajit/pair-matching.

### 4.5. Calculation of JSD values

#### 4.5.1. Calculation of $\text{JS}{\text{D}}^{\left(I_{T}\right)}$

For a subset of *m* molecular species (*m* ≤ *n*), we calculated the sets $\left\{{I_{T}}^{\left(\alpha \right)}(t_{1})\right\}$and $\left\{{I_{T}}^{\left(\beta \right)}(t_{2})\right\}$ for *N* = 3000 cells. We then constructed the probability distributions *P*(*I*_T_, *t*_1_) and P(*I*_T_, *t*_2_) by binning the above sets and normalizing the histograms. The bin width (Δ*I*_T_) was chosen to be the cardinality *k* of the subset. We then calculated the Kullback–Leibler divergence (*d*_KL_) using the distributions. Here *d*_KL_ is given by an integral as *I*_T_ is a continuous variable. We approximated the integral by a sum over the histograms calculated above, i.e.

$$\int_{I_{T}^{\min}}^{I_{T}^{\max}}P(I_{T})\ln\left[\frac{P(I_{T})}{M(I_{T})}\right]\;\text{d}I_{T}\approx \Delta I_{T}\sum_{p=I_{T}^{\min}}^{I_{T}^{\max}}P({\left(I_{T}\right)}_{p})\ln\left[\frac{P({\left(I_{T}\right)}_{p})}{M({\left(I_{T}\right)}_{p})}\right],$$

where Δ*I*_T _= *k* and *M*(*I*_T_)  =  [*P*(*I*_T_(*t*_1_))  +  *P*(*I*_T_(*t*_2_))]/2.

#### 4.5.2. Calculation of $\text{JS}{\text{D}}^{\left(I_{M}\right)}$

Done in a similar way as *I*_T_. The bin width (Δ*I*_M_) was chosen as follows. We calculated Δ*I*^(α)^_M_ following,

$$\Delta {I^{\left(\alpha \right)}}_{M}=\frac{\sum_{i.j}\left(\Delta {x^{\left(\alpha \right)}}_{i}\;{J^{-1}}_{ij}{x^{\left(\alpha \right)}}_{j}\;+{x^{\left(\alpha \right)}}_{i}\;{J^{-1}}_{ij}\Delta {x^{\left(\alpha \right)}}_{j}\right)}{2|{\tilde{x}}^{\left(\alpha \right)}|}=\frac{\sum_{i,j}\left(\Delta {x^{\left(\alpha \right)}}_{i}\;{J^{-1}}_{ij}\;x^{\left(\alpha \right)}{{\;}_{j}}_{j}\;\right)}{\left|{\tilde{x}}^{\left(\alpha \right)}\right|}.$$

Using $I_{M}^{\left(\alpha \right)}=|{\tilde{x}}^{\left(\alpha \right)}|=\sqrt{\sum_{i,j}{x_{i}}^{\left(\alpha \right)}\;{J_{ij}}^{-1}\;x{{\;}_{j}^{\;}}^{\left(\alpha \right)}},$ and $\Delta x^{\left(\alpha \right)}$ = 1 for all species and all the cells the above expression simplifies to

$$\Delta I_{M}^{\left(\alpha \right)}=\frac{\sum_{i,j}J_{ij}^{-1}x_{j}^{\left(\alpha \right)}}{\left|{\tilde{x}}^{\left(\alpha \right)}\right|}.$$

We evaluated $\Delta I_{M}^{\alpha }$ for all the cells at time *t*_1_ and *t*_2_ and set $\Delta I_{M}=\text{min}(\Delta I_{M}^{\left(\alpha \right)})$. A pseudo-code is provided in the electronic supplementary material. The Mathematica codes are available at http://planetx.nationwidechildrens.org/~jayajit/pair-matching.

## Supplementary Material

Supplementary Material for “Connecting the dots across time: Reconstruction of single cell signaling trajectories using time-stamped data”

## Data accessibility

The computer codes used for the studies shown in the article are available at the website: http://planetx.nationwidechildrens.org/~jayajit/pair-matching. The codes are written in Mathematica and in BIONETGEN (bionetgen.org). The live-cell imaging data reported in fig. 4 in [18] are available on the web at the link: http://msb.embopress.org/content/9/1/704.short.
