# Supplementary material for: Connecting the dots across time: reconstruction of single-cell signalling trajectories using time-stamped data
Source: R Soc Open Sci. 2017 Aug 23;4(8):170811. doi: 10.1098/rsos.170811 (PMC5579131; doi:10.1098/rsos.170811)
Supplement: Supplementary Material for “Connecting the dots across time: Reconstruction of single cell signaling trajectories using time-stamped data” [file rsos170811supp1.pdf]

Supplementary Material for "Connecting the dots across time: Reconstruction of single cell signaling trajectories using time-stamped data"

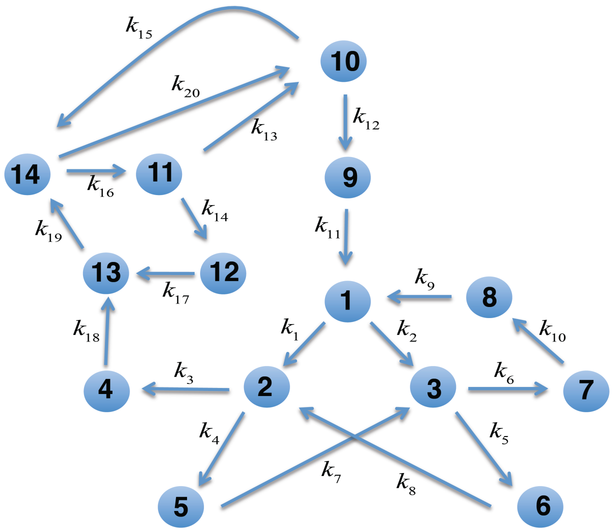

Figure S1: Wiring diagram of the network of first order reactions. The species are indicated by the integers and the rate constants shown as $\{k_a\}$. The values for the rate constants and the initial abundances are shown in Table S1.

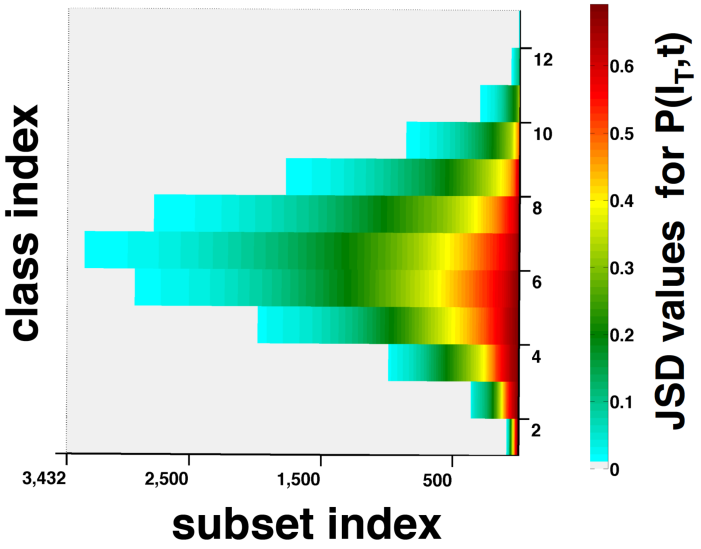

Figure S2: Variation of $JSD^{(I_T)}$ with subsets in the linear network. The calculation was carried out for the 16369 different subsets of signaling species involving 3000 single cells at a pair of time points (t_1=0, t_2=7min). The kinetics is described by first order reactions corresponding to the network in Fig. S1 and Table S1.

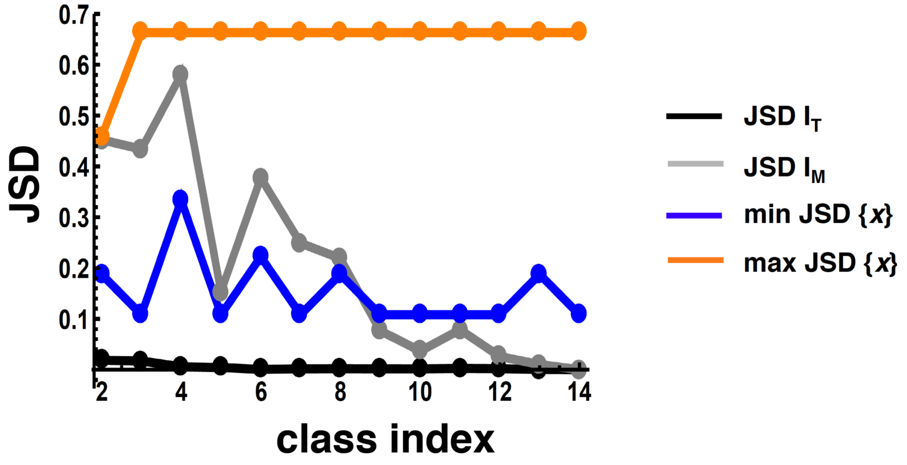

Figure S3: I_T behaves as a slow variable or an invariant in the linear kinetics. Shows the minimum values of $JSD^{(I_T)}$ for each class (black points) for the kinetics with first order reactions. The parameter values are the same as in Fig. S2. JSD values associated with the fastest (shown in orange) and the slowest species (shown in blue) in the subsets corresponding to the minimum $JSD^{(I_T)}$ are compared with minimum $JSD^{(I_T)}$. The grey line shows the values of $JSD^{(I_M)}$ for the subsets that yielded the min($JSD^{(I_T)}$). For multiple subsets (e.g., the subsets corresponding to class#10, #12, #13) , both I_T and I_M behave as slow variables.

(A)

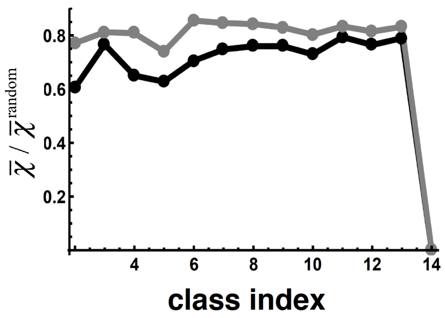

(B)

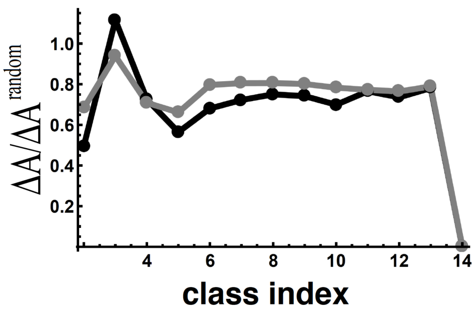

Figure S4: Result of pairing using I_T for the linear kinetics. The reconstruction was carried out using I_T for the subsets that produced min $\text{JSD}^{(I_T)}$ values (Fig. S3). **(A)** The ratio (black points) of the average error in the reconstruction with that for random pairing corresponding to subsets. The grey points show the error when the reconstruction was carried out for the same subsets using I_M instead of I_T. (B) Shows error in the autocorrelation function for the same reconstructions in (A).

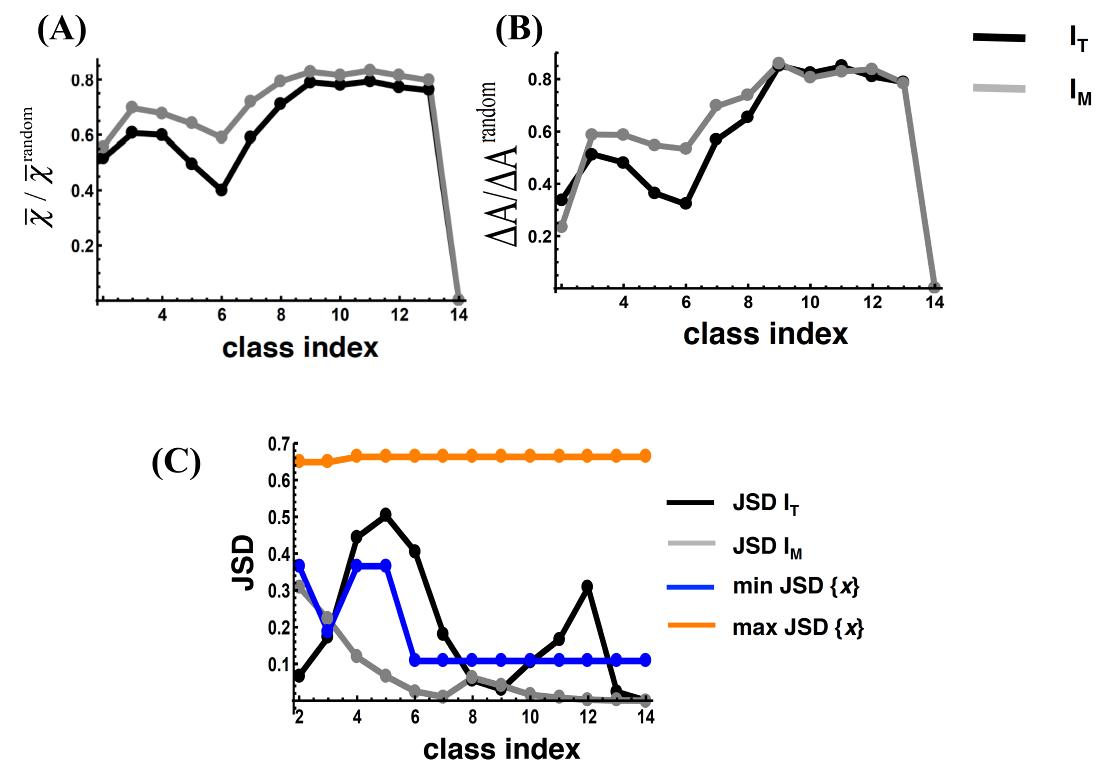

Figure S5: Comparison of the reconstructions carried out using I_M or I_T for the linear network. The reconstruction was carried out for the subsets that produced minimum $JSD^{(I_M)}$. The parameters are the same as in Fig 2. (A) Shows the error (grey points) when the reconstruction was performed using I_M. The black points show the errors when the single cells were paired using I_T for the same subsets that yielded minimum $JSD^{(I_M)}$. (B) Shows error in the autocorrelation function for the reconstructions in (A). The pairings using I_T show lower error even though the subsets produced minimum values of $JSD^{(I_M)}$. (C) Shows the $JSD^{(I_T)}$ values (black points) for the subsets that produced minimum values of $JSD^{(I_M)}$. In several cases (e.g., subset for class #4) I_T has a faster kinetics than I_M.

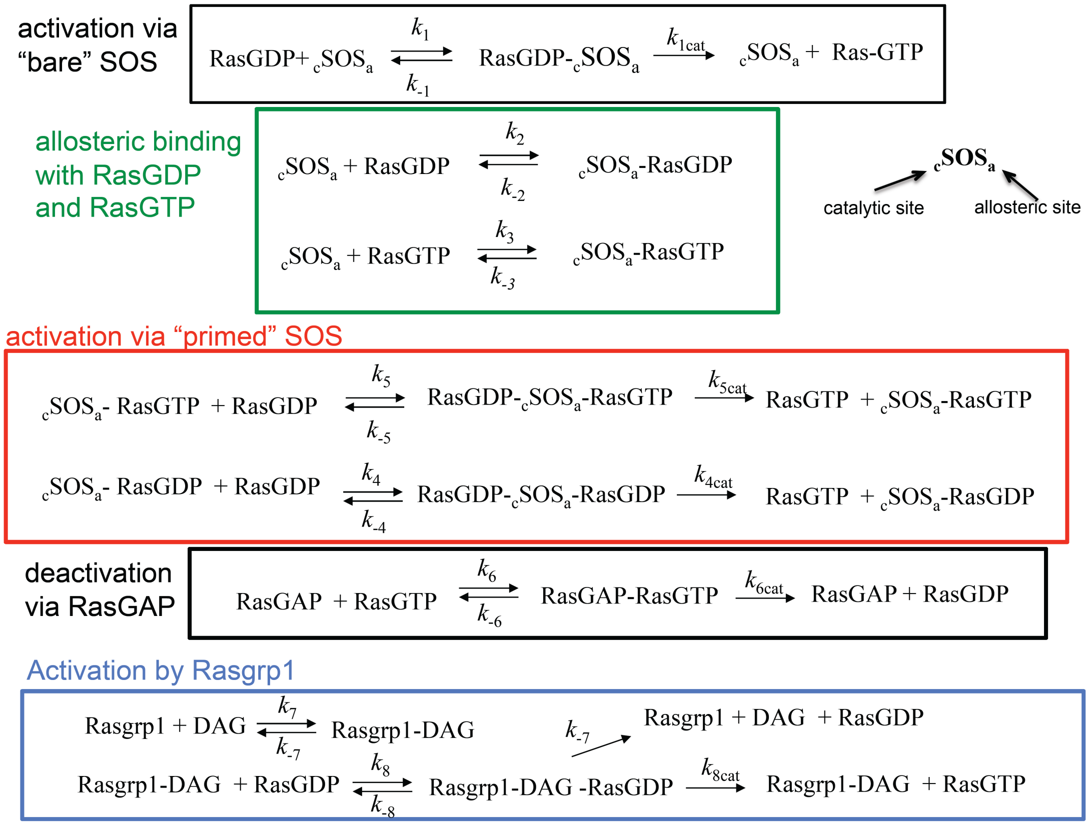

Figure S6: The Ras activation network. SOS and Rasgrp1 are the enzymes that convert the de-active form of Ras or RasGDP to the active form, RasGTP. SOS binds RasGDP at the catalytic site and an allosteric site. In addition, the allosteric site in SOS can bind to RasGTP. When the allosteric site of SOS is occupied by RasGTP or RasGDP, the catalytic rate for Ras activation is increased. This creates a positive feedback in the activation. Rasgrp, when bound to the membrane bound DAG, acts as an enzyme for Ras activation. Ras de-activation is carried out by the enyme RasGAP. The reactions and the rates are shown above. The rates and initial conditions are shown in Table S2.

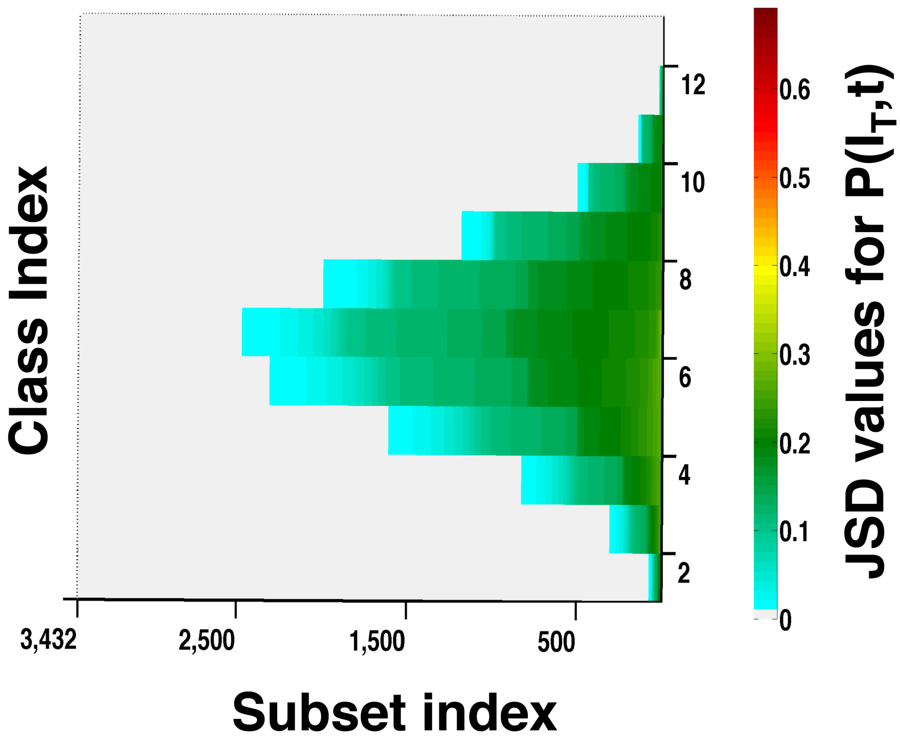

Figure S7: Variation of $JSD^{(I_T)}$ for the deterministic Ras activation kinetics. We used 3000 single cells across time points t_1=100s and t_2=400s where the Ras activation displays bistability. The kinetics was simulated using the reactions shown in Fig. S6 using the software package BIONETGEN.

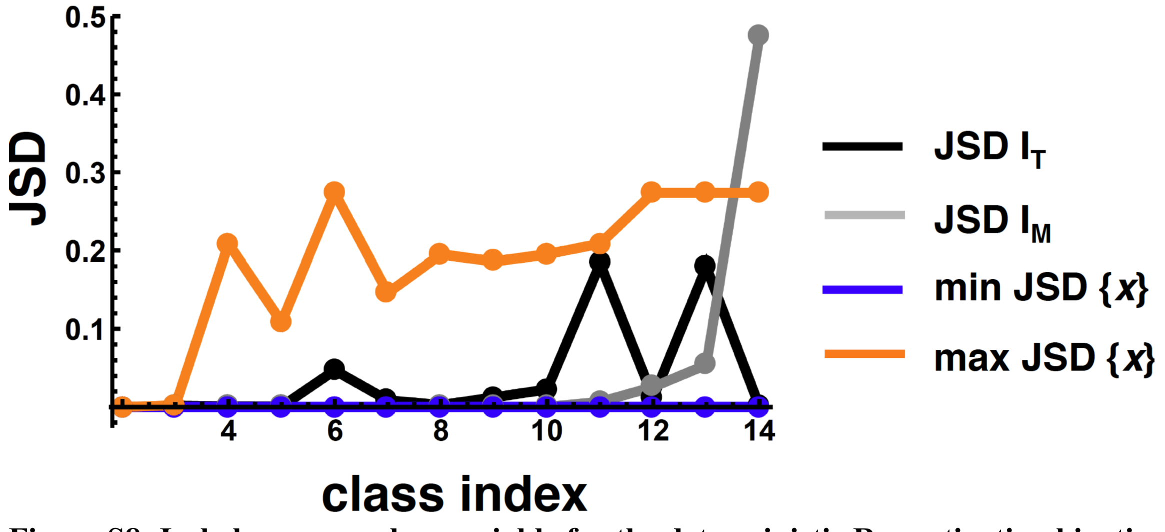

Figure S8: I_M behaves as a slow variable for the deterministic Ras activation kinetics. Shows the minimum values of $JSD^{(I_M)}$ (grey points) for all the subsets. The JSD values corresponding to the fastest (orange points) and the slowest species (blue points) are

shown for comparison. Some of the species abundances reached values closer to the steady state and generate very small change in the time window. When present, these species corresponded to the slowest species. $JSD^{(I_M)}$ values were equal (very close to zero) to the JSD values for the slowest species for the subsets until class#10. Thus, I_M behaved as slow variables in these subsets. $JSD^{(I_T)}$ values for the subsets generating minimum $JSD^{(I_M)}$ are shown in black.

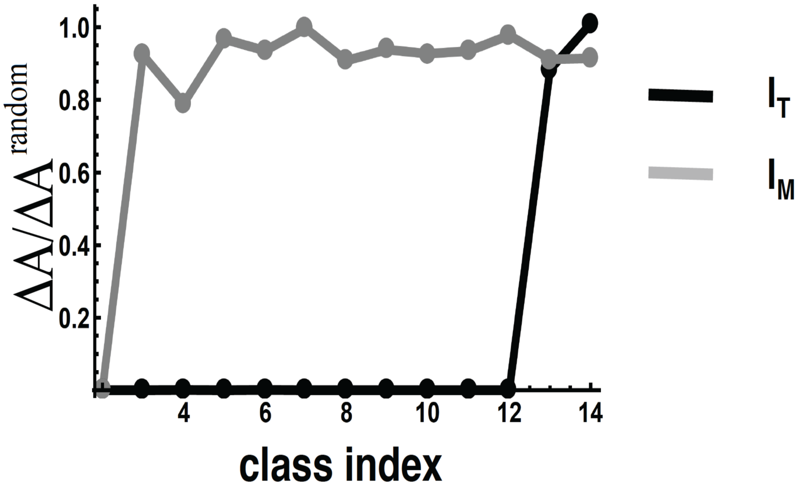

Figure S9: Error in the autocorrelation for the pairings performed using I_T and I_M for the deterministic Ras activation kinetics. Error in the autocorrelation function when the pairings were performed using I_T and I_M. The pairings were carried out for the subsets that yielded minimum values for $JSD^{(I_T)}$ (black) or $JSD^{(I_M)}$ (grey). The subsets are shown with their class indices. Note, for each class the subsets used for I_T or I_M are different. The data compare the best level of pairings that can be carried out by I_T or I_M. Except the subsets with 2, 13, and, 14 species (class #2, #13, and #14, respectively), pairing by I_T performs substantially better than that by I_M.

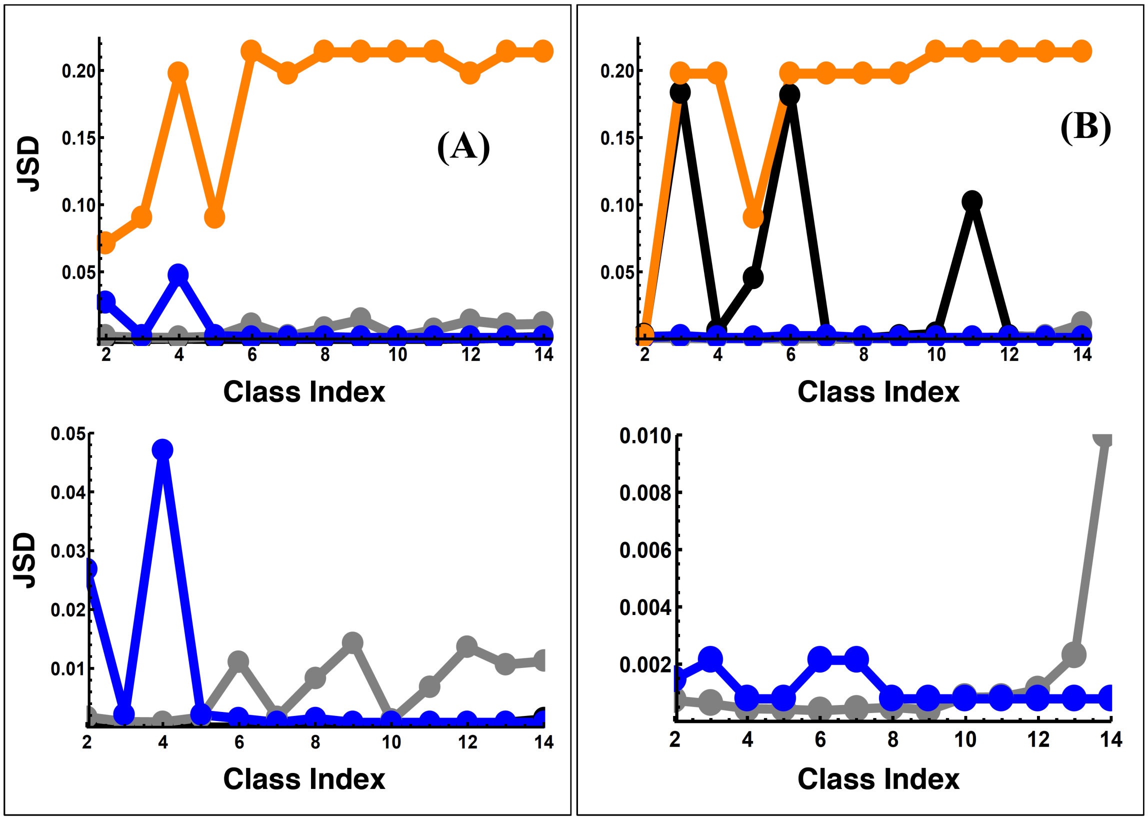

Figure S10: I_T and I_M behaves as a slow variable for stochastic Ras activation kinetics. Ras activation kinetics including intrinsic noise fluctuations was generated using the same reaction network used for investigating the deterministic kinetics. The simulations were carried out using the software package BIONETGEN. The parameters used for the kinetics are the same as that used for Fig. S8. **(A)** Shows the minimum values of $JSD^{(I_T)}$ (black points) for all the subsets. The values for $JSD^{(I_T)}$ are very close to zero and are covered by the grey or the blue symbols. The JSD values corresponding to the fastest (orange points) and the slowest species (blue points) are shown for comparison. The $JSD^{(I_M)}$ values corresponding to the subsets for min ($JSD^{(I_T)}$) are shown in grey. The separation between the points at the lower JSD values are shown in the bottom panel where the y axis is zoomed in between 0 to 0.05. **(B)** Shows the minimum values of $JSD^{(I_M)}$ (grey points) for all the subsets. The JSD values corresponding to the fastest (orange points) and the slowest species (blue points) are shown for comparison. The $JSD^{(I_T)}$ values corresponding to the subsets for min ($JSD^{(I_M)}$) are shown in black. The bottom panel shows the zoomed in version of the top graph (grey and blue points only) for the points at the lower JSD values.

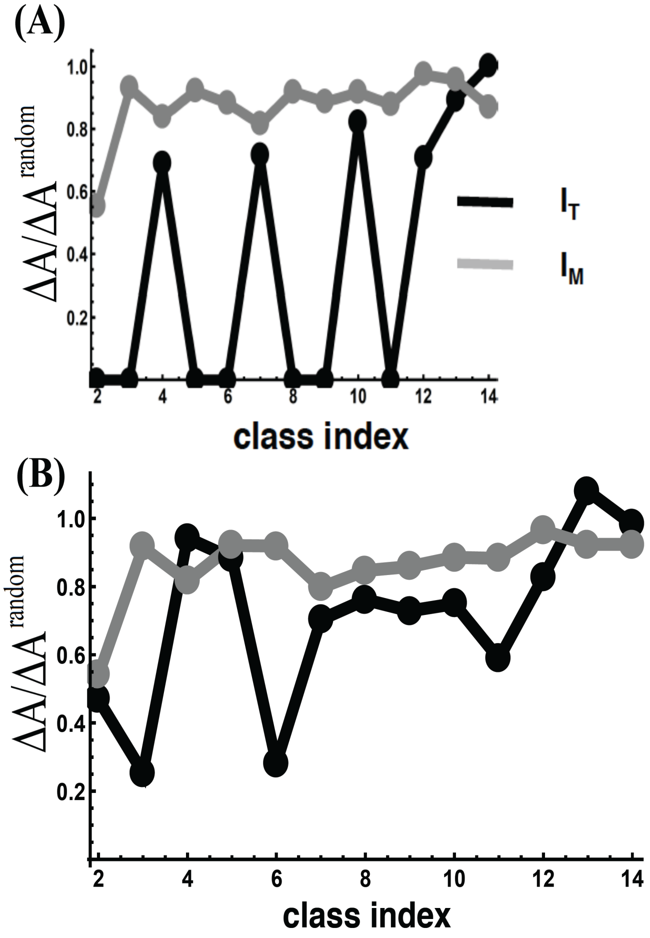

Figure S11: Error in the autocorrelation function for the pairings performed using I_T and I_M for the stochastic Ras activation kinetics. The pairings were carried out for 3000 single cells for stochastic Ras activation kinetics. **(A)** Pairing was done using I_T for the subsets (indexed by the class numbers) that yielded minimum values of $JSD^{(I_T)}$. Error in the autocorrelation function (black points) for single cell-sister cell pairs was calculated for the same subset. The grey points show the error when I_M was used for pairing the single cells for the same subsets that yielded minimum values of $JSD^{(I_T)}$. **(B)** Pairing was done using I_M for the subsets (indexed by the class numbers) that yielded minimum values of $JSD^{(I_M)}$. Error in the autocorrelation function (grey points) for single cell-sister cell pairs was calculated for the same subset. The black points show the error when I_T was used for pairing the single cells for the same subsets that yielded minimum values of $JSD^{(I_M)}$.

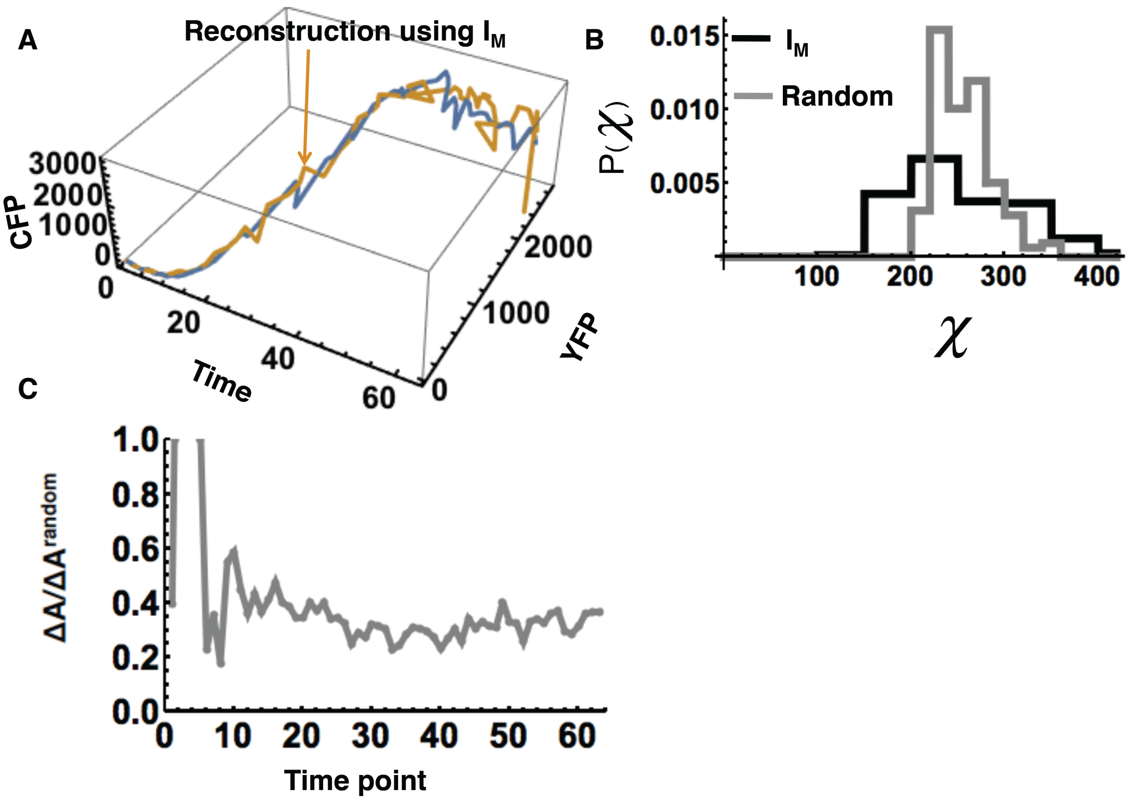

Figure S12: Quantification of errors in reconstructed trajectories using I_M in live-cell imaging. The live cell imaging data as described in Fig. 5 was used to generate reconstructed trajectories. We used 159 single cell trajectories for the analysis. **(A)** Shows the reconstructed trajectory for one cell using I_M. **(B)** Distribution of the error χ , $P(\chi)$ (in black), for the reconstruction carried out using I_M. $P(\chi)$ for random pairing is shown in grey for comparison. **(C)** Error in the autocorrelation function in the reconstructed trajectories by I_M. The autocorrelation was calculated for the successive pairs of time points (e.g., 0min to 2.5min, 2.5min to 5min, and so on) available in the data. For most of the time points we find $\Delta A/\Delta A_{random} < 1$.

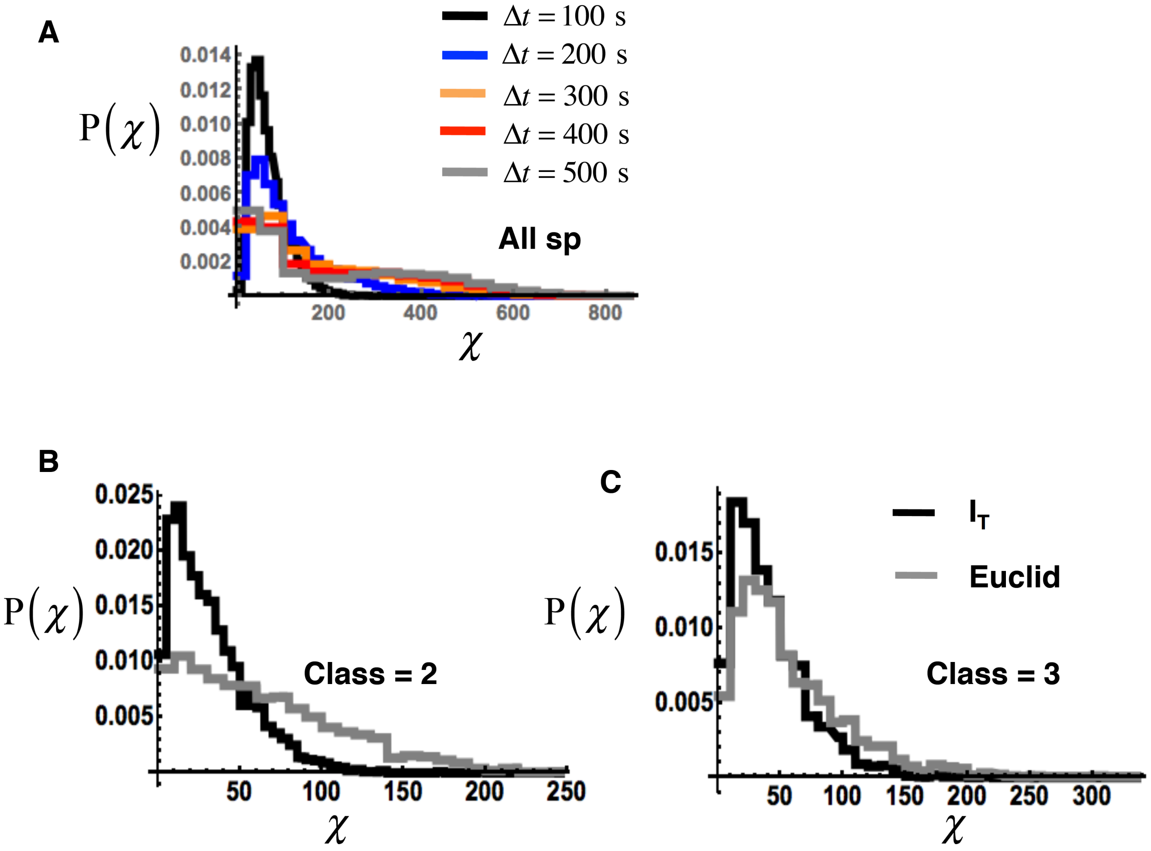

Figure S13: (A) Comparison of the distribution of χ when the time difference between time stamped data for a bistable Ras-SOS network is progressively increased. 6 species namely SOS, RasGDP, RasGTP, RasGAP, DAG and RasGRP were assayed at times t=0 to t=500 s with an increment of 100 s between successive assays. The solid lines in black, blue, orange, red and grey show P(χ) for the reconstructions for data sets assayed at time t=0 and t= 100s, t=0 and t=200 s, t=0 and t=300s, t=0 and t=400 s and t=0 and t=500 s respectively. The system approach the bistable Ras activation close to t=300s. **(B)** Shows the distribution of the reconstruction error χ carried out for the subset that produced minimum $JSD^{(I_T)}$ for k=2 for the data set used in Fig. 6 in the main text. The reconstructions were carried out using I_T (black) or with a method that minimizes the total Euclidean distance (grey). **(C)** Same as (B) except the comparison is done for the subset with minimum $\mathrm{JSD}^{(I_T)}$ for k=3.

Table S1A: Rate constants for the network in Fig. S1

**Rate constants**	**min^{-1}**
k_1	0.08
k_2	0.1
k_3	0.145
k_4	0.226
k_5	0.321

k_6	0.178
k_7	0.134
k_8	0.245
k_9	0.48
k_{10}	0.50
k_{11}	0.033
k_{12}	0.23
k_{13}	0.128
k_{14}	0.67
k_{15}	0.45
k_{16}	0.51
k_{17}	0.11
k_{18}	0.05
k_{19}	0.32
k_{20}	0.20

Table S1B: Initial conditions for the network in Fig. S1

The initial species copy numbers are drawn from a multivariate Gaussian distribution. Below we quote the average values and covariance matrix for the species abundances.

**Average**	**# of molecules**
Species 1	122
Species 2	186
Species 3	192
Species 4	259
Species 5	101
Species 6	268
Species 7	176
Species 8	196
Species 9	209
Species 10	158
Species 11	173
Species 12	225
Species 13	286
Species 14	202

Covariance Matrix

1326.54	-32.6209	441.557	521.367	23.646	283.172	-143.846	314.964	222.966	218.515	-61.7517	486.626	-594.715	-420.437
-32.6209	3153.51	65.5223	-84.8321	-12.8702	810.822	-267.412	679.462	439.157	-274.004	30.4547	-62.6026	-219.974	301.26
441.557	65.5223	3376.05	-29.3363	224.051	938.333	-377.637	52.7194	-250.807	83.6003	-318.352	-367.788	8.1994	13.5782
521.367	-84.8321	-29.3363	5902.05	44.8606	-7.75098	579.177	-579.456	488.745	349.197	-801.413	-986.762	1479.98	899.332
23.646	-12.8702	224.051	44.8606	939.701	-496.84	74.7393	167.398	-152.286	233.251	131.992	424.503	-212.305	88.9822
283.172	810.822	938.333	-7.75098	-496.84	6374.07	-592.042	-194.304	307.236	107.392	-298.845	669.602	657.67	-198.722
-143.846	-267.412	-377.637	579.177	74.7393	-592.042	2738.85	-306.283	-396.695	139.609	381.185	-39.9622	-147.113	-164.373
314.964	679.462	52.7194	-579.456	167.398	-194.304	-306.283	3436.46	730.01	474.362	539.552	-289.618	-1040.05	-422.465
222.966	439.157	-250.807	488.745	-152.286	307.236	-396.695	730.01	3926.89	33.2891	71.2203	-338.489	-753.413	421.906
218.515	-274.004	83.6003	349.197	233.251	107.392	139.609	474.362	33.2891	2246.67	-145.769	440.031	-97.3797	342.974
-61.7517	30.4547	-318.352	-801.413	131.992	-298.845	381.185	539.552	71.2203	-145.769	2733.75	655.68	-855.245	554.293
486.626	-62.6026	-367.788	-986.762	424.503	669.602	-39.9622	-289.618	-338.489	440.031	655.68	4528.02	788.084	-636.284
-594.715	-219.974	8.1994	1479.98	-212.305	657.67	-147.113	-1040.05	-753.413	-97.3797	-855.245	788.084	7414.02	951.054
-420.437	301.26	13.5782	899.332	88.9822	-198.722	-164.373	-422.465	421.906	342.974	554.293	-636.284	951.054	3677.34

Table S2A: Rate constants for the Ras activation kinetics network in Fig. S6

Rate Constants	
k_1	0.0053 $\mu M^{-1}s^{-1}$
k_{-1}	4.0 s^{-1}
k_{1cat}	0.0005 s^{-1}
k_2	0.12 $\mu M^{-1}s^{-1}$
k_{-2}	3.0 s^{-1}
k_3	0.11 $\mu M^{-1}s^{-1}$
k_{-3}	0.4 s^{-1}
k_4	0.07 $\mu M^{-1}s^{-1}$
k_{-4}	1.0 s^{-1}
k_{4cat}	0.003
k_5	0.05 $\mu M^{-1}s^{-1}$
k_{-5}	0.1 s^{-1}
k_{5cat}	0.038
k_6	1.74 $\mu M^{-1}s^{-1}$
k_{-6}	0.2 s^{-1}
k_{6cat}	0.1 s^{-1}
k_7	0.1 $\mu M^{-1}s^{-1}$
k_{-7}	5.0 s^{-1}
k_8	0.33 $\mu M^{-1}s^{-1}$
k_{-8}	1.0 s^{-1}
k_{8cat}	0.01 s^{-1}

Cytosolic volume used =0.08 μm^3, surface area and the depth of plasma membrane used are 4.0 μm^2 and 1.7 nm respectively.

Table S2B: Copy numbers for the species used in the Ras activation network in Fig. S6

The initial copy numbers of the molecular species are drawn from a multivariate Gaussian distribution. Below we show the average values and the covariance used. The species that are not shown here have zero abundances at t=0.

**Average**	**# of molecules**
#1. Sos	85
#2. RasGDP	370
#3. RasGTP	30
#4. RasGap	10
#5. RasGRP1	47
#6. DAG	40

Covariance matrix.

$$\begin{pmatrix} 634.324 & 534.672 & 10.0372 & 12.9434 & 50.9679 & 41.6175 \\ 534.672 & 12269.9 & 115.168 & 14.3392 & 343.09 & -111.897 \\ 10.0372 & 115.168 & 79.1503 & 4.11305 & 21.4583 & 9.31962 \\ 12.9434 & 14.3392 & 4.11305 & 9.10811 & -6.54498 & -3.41377 \\ 50.9679 & 343.09 & 21.4583 & -6.54498 & 209.92 & -12.0996 \\ 41.6175 & -111.897 & 9.31962 & -3.41377 & -12.0996 & 138.968 \end{pmatrix}$$

Pseudocodes for the algorithms used for implementing the framework

Notations

N: Number of species measured

$k \subset N$ {k=2, 3, …, N}: Class with k species

m {m=1, 2, 3, …, $^{N}C_{k}$}: sub-modules with k species (elements of class k)

$[D_{t1}^{m,k}]_{ij}$ {i=1, 2, ….., # of cells}, {j=1, 2, .., k}: Data matrix at time t_1 of the mth sub-module belonging to class k. For example $[D_{t1}^{1,2}]_{ij}$ contains first 2 species at time t_1 whereas $[D_{t1}^{2,2}]_{ij}$ contains the first and the third species at time t_1.

$[D_{t2}^{m,k}]_{ij}$ {i=1, 2, ….., # of cells}, {j=1, 2, .., k}: Same as $[D_{t1}^{m,k}]_{ij}$ except the species abundances are measured at time t_2.

$[A_{t2}^{m,k}]_{ij}$ {i=1, 2, ….., # of cells}, {j=1, 2, .., k}: Data matrix for the sub-module m belonging to the class k at time t_2 that contains the species abundance of the correct partners of the cells in $[D_{t1}^{m,k}]_{ij}$. So if correctly aligned the first cell (row #1) in $[D_{t1}^{m,k}]_{ij}$ would be paired to the first cell (row #1) of $[A_{t2}^{m,k}]_{ij}$.

M^{JSD}_{ij} {i=1, 2, …, N-1}, {j=1, 2}: Array for the min JSD values for each class $k \in$ {2, 3, …, N}.

$[I_T^{t1}]_i = \sum_{j=1}^{k} \left[D_{t1}^{m,k} \right]_{i,j}$ {i=1, 2, # of cells}: Array containing sum of the species abundances of the mth sub-module belonging to class k at time t_1.

$[I_T^{t2}]_i = \sum_{j=1}^{k} \left[D_{t2}^{m,k} \right]_{i,j}$ {i=1, 2, # of cells}: Array containing sum of the species abundances of the mth sub-module belonging to class k at time t_2.

B_{ij} {i=1, 2, …., # of cells}, {j=1, 2}: Alignment matrix. If the first row of B is {3, 4} then it means that the 3rd cell at time t_1 (3rd row in $[D_{t1}^{m,k}]$) is aligned to the 4th cell at time t_2 (4th row in $[D_{t2}^{m,k}]$).

$QAlg_i$ {i=1,N-1}: Array for the quality of alignment

Algorithm for minimum JSD

Input: $D_{t1}^{m,k}$, $D_{t2}^{m,k}$

Output: M^{JSD}

```
Initialize M^JSD_{i,1}=α where α > ln2 ∀ i ∈{1, N-1}.
 for k ← 2 to N do
        for m ← 1 to ^N C_k do
                load D_t1^{m,k} and D_t2^{m,k}
                calculate I_T^t1 and I_T^t2
                calculate P= distribution of I_T^t1 and
                         Q= distribution of I_T^t2
               calculate JSD(P, Q) using eqn (2.7) main text
               If JSD(P, Q) < M^JSD_{k-1,1} then
```

$M^{JSD}_{k\text{-}1,1}$ = JSD(P, Q) and $M^{JSD}_{k\text{-}1,2} = m$

endif

end

end

Print M^{JSD}

Algorithm for calculation of quality of reconstruction

Input: M^{JSD}, $D_{t1}^{m=m^*,k}, D_{t2}^{m=m^*,k}, A_{t2}^{m=m^*,k}$ where m^* denotes the sub-module with minimum JSD

Ouput: QAlg

for $k \leftarrow 2$ to N do

Load $D_{t1}^{m^*,k}, D_{t2}^{m^*,k}, A_{t2}^{m^*,k}$ using M^{JSD}

for $i \leftarrow 1$ to # of cells do

build matrix T1 and T2 such that

$T1_{i,1} = i$ and $T1_{i,2} = \sum_{j=1}^{k} \left[D_{t1}^{m^*,k} \right]_{ij}$ and

$T2_{i,1} = i$ and $T2_{i,2} = \sum_{j=1}^{k} \left[D_{t2}^{m^*,k} \right]_{ij}$

end

build ST1= Sort T1 by column 2

ST2= Sort T2 by column 2

sum1=0

sum2=0

for $i \leftarrow 1$ to # of cells do

$B_{i,1} = ST1_{i,1}$

$B_{i,2} = ST2_{i,1}$

$$\text{sum1} = \text{sum1} + \sqrt{\sum_{j=1}^{k} \left(A_{B_{i,1},j} - \left[D_{t2}^{m^*,k} \right]_{B_{i,2},j} \right)^2}$$

$$\text{sum2} = \text{sum2} + \sqrt{\sum_{j=1}^{k} \left(A_{B_{i,1},j} - \left[D_{t2}^{m^*,k} \right]_{Rand[1,\#\text{ of cells}],j} \right)^2}$$

end

```
        QAlg_{k-1}=sum1/sum2
end
```

Physical interpretation of the invariant I_M

The time evolution changes the abundance vector $\vec{x}^{(\alpha)}(t)$ as

$$x_i^{(\alpha)}(t_2) = \left[e^{M(t_2-t_1)}\right]_{ij} x_j^{(\alpha)}(t_1) = A_{ij}(t_2,t_1)x_j^{(\alpha)}(t_1) \quad \text{(S1)}$$

A is an n×n real matrix. Suppose, the vectors, $\vec{x}^{(\alpha)}(t_2)$ and $\vec{x}^{(\alpha)}(t_1)$, are scaled by matrices S_2 and S_1, respectively, such that the change given by Eq. (S1) transforms the scaled vectors (denoted by, $\vec{\tilde{x}}^{(\alpha)}(t_2)$ and $\vec{\tilde{x}}^{(\alpha)}(t_1)$, respectively) by an orthogonal matrix, i.e., the scaled vectors undergo rotation or reflection. The scaled vectors are given by,

$$\vec{\tilde{x}}^{(\alpha)}(t_2) = S_2.\vec{x}^{(\alpha)}(t_2) \quad \text{(S2)}$$
$$\vec{\tilde{x}}^{(\alpha)}(t_1) = S_1.\vec{x}^{(\alpha)}(t_1)$$

The "." operation in the above equations denotes matrix multiplication. Eq. (S1) can be written in terms of the scaled variables as,

$$\vec{\tilde{x}}^{(\alpha)}(t_2) = S_2.A.S_1^{-1}.\vec{\tilde{x}}^{(\alpha)}(t_1) \quad \text{(S3)}$$

The matrix, S_2A can be polar decomposed(1) as,

$$S_2A = QP$$

where, Q is an orthogonal matrix ($QQ^T=I$) and

$$P = \sqrt{(S_2A)^T(S_2A)} \ .$$

Below we show that when $S_2=[J(t_2)]^{-1/2} =J_2^{-1/2}$ and $S_1=[J(t_1)]^{-1/2}=J_1^{-1/2}$, then $S_2AS_1^{-1}$ in Eq. (S3) is equal to the orthogonal matrix Q.

$$S_2.A.S_1^{-1} = QPS_1^{-1} = Q\sqrt{(S_2A)^T(S_2A)}S_1^{-1} = Q\sqrt{(J_2^{-1/2}A)^T(J_2^{-1/2}A)}J_1^{1/2} = Q\sqrt{(A^TJ_2^{-1}A)}J_1^{1/2}$$

According to Eq. (15b) in the main text, $J_2=AJ_1A^T$ or $J_2^{-1}=(A^T)^{-1}(J_1)^{-1}A^{-1}$ or $A^T J_2^{-1} A=(J_1)^{-1}$. As a result,

$$S_2.A.S_1^{-1} = Q\sqrt{(A^TJ_2^{-1}A)}J_1^{1/2} = QJ_1^{-1/2}J_1^{1/2} = Q \ .$$

Therefore, scaling the abundance vector (Eq. (S2) or Eq. (3) in the main text) makes the time evolution of the scaled vectors either a rotation or a reflection. As a result the magnitude of the scaled vector (or I_M) does not change with time (Eq. (6) in the main text).

1. Halmos PR (1987) *Finite-dimensional vector spaces* (Springer-Verlag, New York) pp viii, 199 p.
